# Transcriptome Analysis of *Gossypium hirsutum* L. Reveals Different Mechanisms among NaCl, NaOH and Na_2_CO_3_ Stress Tolerance

**DOI:** 10.1038/s41598-018-31668-z

**Published:** 2018-09-10

**Authors:** Binglei Zhang, Xiugui Chen, Xuke Lu, Na Shu, Xiaoge Wang, Xiaomin Yang, Shuai Wang, Junjuan Wang, Lixue Guo, Delong Wang, Wuwei Ye

**Affiliations:** State Key Laboratory of Cotton Biology, Institute of Cotton Research of Chinese Academy of Agricultural Sciences, Key Laboratory for Cotton Genetic Improvement, Anyang, 455000 Henan China

## Abstract

As an important source of fiber and edible oil, cotton has great economic value. In comparison to their individual studies, association and differentiation between salt and alkaline tolerance has not been focused yet by scientists. We have used next-generation RNA-Seq technique to analyze transcriptional changes under salt and alkaline stresses in cotton. Overall, 25,929 and 6,564 differentially expressed genes (DEGs) were identified in roots and leaves, respectively. Gene functional annotation showed that genes involving ionic homeostasis were significantly up-regulated under NaCl stress and Na_2_CO_3_ stress, and genes enriched in starch and sucrose metabolism were up-regulated under NaOH stress and Na_2_CO_3_ stress. Furthermore, a synergistic enhancing effect between NaCl and NaOH stress was also observed in this study. Likewise, our studies indicate further that genes related with starch and sucrose metabolism were regulated to respond to the high pH under Na_2_CO_3_ stress, inducing plant hormone signal transduction and key enzyme reactive oxygen species (ROS) activity to respond to ionic toxicity and intracellular ionic homeostasis. By analyzing the expression profiles of diverse tissues under different salt and alkaline stresses, this study provides valuable ideas for genetic improvements of cotton tolerance to salt-alkaline stress.

## Introduction

Plant growth and development is highly influenced by various biotic and abiotic factors, resulting in a destructive impact on their production. In the north east area of china, soil alkalinity is a major abiotic stress which is responsible for the decline of agricultural production and causes environmental hazard. Soils are considered as Saline-Alkaline soils, upon salinity in soils goes above 0.3%^1^.

Soil affected by salt are basically classified in three categories: saline soils, alkaline soils and salt-alkaline soils^1^. Saline soils comprises of excessive amount of neutral salts, which includes NaCl and Na_2_SO_4_, as a major part, resulting in salt stress. NaHCO_3_ and Na_2_CO_3_ are responsible for the alkalization of soils by creating a high pH value, with a destructive effect on plants growth^2^. Stress resulted from alkaline soils causes several issues of osmotic pressure stress, different types of ionic injuries and high pH stress. Plants under salt-alkaline conditions suffer from both salt stress caused by excessive salt ions and alkaline stress caused by high pH^3,4^.

Salt-alkaline stress causes several damage to plants because of the presence of salt ions and high pH. Previous studies have shown that plants maintain intracellular homeostasis through osmotic adjustment, generating an active oxygen scavenging system and adjusting organic acid under NaCl stress^5,6^. Genes associated with brassinosteroid biosynthesis were upregulated under alkaline stress (Na_2_CO_3_)^6,7^. Cao *et al*. found that the expression of *Gshdz4* was induced by NaHCO_3_, indicating that *Gshdz4* is only responsible for resisting HCO_3_^−^, but not high pH^8^. The response to NaHCO_3_ of *T*. *hispida* involves multiple physiological and metabolic pathways^9^. The *Aft2* gene plays a negative role, and Na^+^-ATPase ENA1 is regulated during alkaline pH resistance in *Saccharomyces cerevisiae*^10^. Three major mechanisms of plant resistance to salt stress have been found: maintenance of ionic and osmotic homeostasis, detoxification and growth regulation^11^. High pH can also affect ion balance, root growth^4^, organic acid accumulation^12^ and cellular processes^13^. However, a little is known about the difference between salt and alkaline stress.

As an important source of fiber, vegetable protein and edible oil, cotton has great economic importance^14^. Soil salt-alkalinity has become an increasingly serious factor in cotton yield because of irrigation and secondary salinization^15^. So development of salt-alkaline tolerant cotton is absolutely of indispensable importance. In recent years, much attention was paid to salt tolerance in cotton. However, the alkaline tolerance of cotton has not been well studied. Few research has been conducted to show the different responses to salt and alkaline stress in cotton. Recent studies have showed that genome-wide transcriptome analysis of cotton has become popular for studying stress tolerance^16,17^. Because the genes involved in salt-alkaline response are numerous, salt-alkaline genes can be more easily identified via transcriptome and DEG analyses.

In our study, we used RNA-seq technology to analyze the expression profiles of diverse tissues under different salt and alkaline stresses. Firstly, the phenotypic changes in roots and leaves of cotton under Na_2_CO_3_, NaCl, and NaOH stress were observed, and the physiological indexes were measured to demonstrate phenotypic differences. Secondly, we studied the transcriptome changes between the stress groups and the control group. Finally, the difference between salt and alkaline stress was obtained. This study provides new ideas for genetic improvement of cotton tolerance to salt-alkaline stress.

## Results

### Phenotypic and physiological responses to different salt-alkaline stresses in *Gossypium hirsutum* L

**Figure 1:**
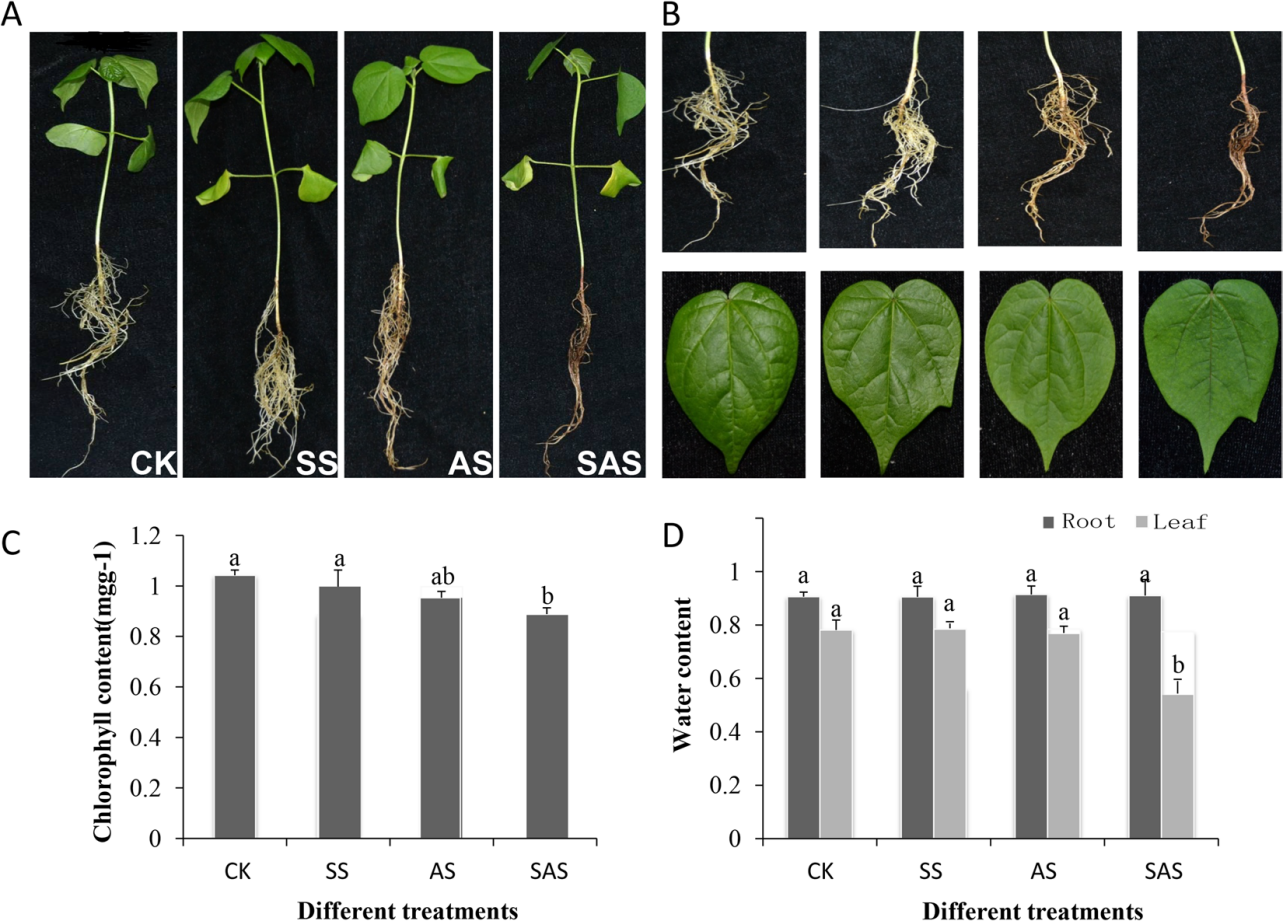
Phenotypic changes of plants under different treatments and measurement of biological indicators. SS: 100 mM NaCl; AS: 0.125 mM NaOH; SAS: 50 mM Na_2_CO_3_. (**A**) Phenotypic changes of plants when Zhong9807 was under the SS, AS and SAS treatments. (**B**) Phenotypic changes in roots and leaves. (**C**) Chlorophyll content under different stress treatments. (**D**) Water content of roots and leaves under different stress treatments.

Previous studies have reported that cotton is more sensitive to abiotic stresses at three-leaf stage^18^. Different morphological has been observed in *G*. *hirsutum* Zhong 9807 during its three-leaf stage under various concentrations of Na_2_CO_3_ stress. We found that seedlings became obviously different between the treatment and control groups under 50 mM Na_2_CO_3_ stress after 12 h (Fig. S1). Two treatments with parallel concentrations of 100 mM and 0.125 mM for NaCl and NaOH respectively were considered to study the comparative effects of CO_3_^2−^, Na^+^ and high pH (Table 1). The salt-alkaline tolerance of Zhong 9807 was examined by comparing salt stress (NaCl stress) with two other alkaline stresses, NaOH stress and Na_2_CO_3_ stress. The phenotypic changes of the three treatments were shown (Fig. 1A,B). Leaves withered and lost lustre slightly under NaCl stress, while roots have no evident changes in phenotype, suggesting roots may have stronger resistance than leaves. However, slight tarnish in leaves and nigrities were found in roots under NaOH stress. When seedlings were subjected to Na_2_CO_3_ stress, roots became seriously withered and nigrescent, leaves tarnished heavily and veins almost darkened. The variance analysis results^19^ of the chlorophyll content and relative water content (RWC) under Na_2_CO_3_ stress (Fig. 1C,D) were significantly different from those of the control group except the RWC in roots.

**Table 1:** Stress comparison for 50 mM Na_2_CO_3_ with 100 mM NaCl and 0.125 mM NaOH.

Component	Na_2_CO_3_	NaCl	NaOH
Na^+^ (mM)	100	100	0.125
pH	11.32	7.00	11.32

### Transcriptome sequencing and alignment

Using the allotetraploid species *Gossypium hirsutum* Zhong9807, RNA-Seq analyses were conducted on three biological repetitions of each sample. RNA samples of roots and leaves were collected at 12 h post-salt stress and alkaline stress. Seedlings transplanted to normal conditions were used as controls. 24 qualified libraries were established (Table S1). Raw reads were processed to remove adapter and low-quality reads initially. Clean reads were then mapped to the *G*. *hirsutum* referenced genome using TopHat2^20^. Approximately 213.36 Gb of clean reads was obtained. On average, 6.15 Gb of clean reads was obtained from each library. More than 87.02% of reads’ Q-score was Q30, and 78.23–83.44% of the total reads were aligned.

**Figure 2:**
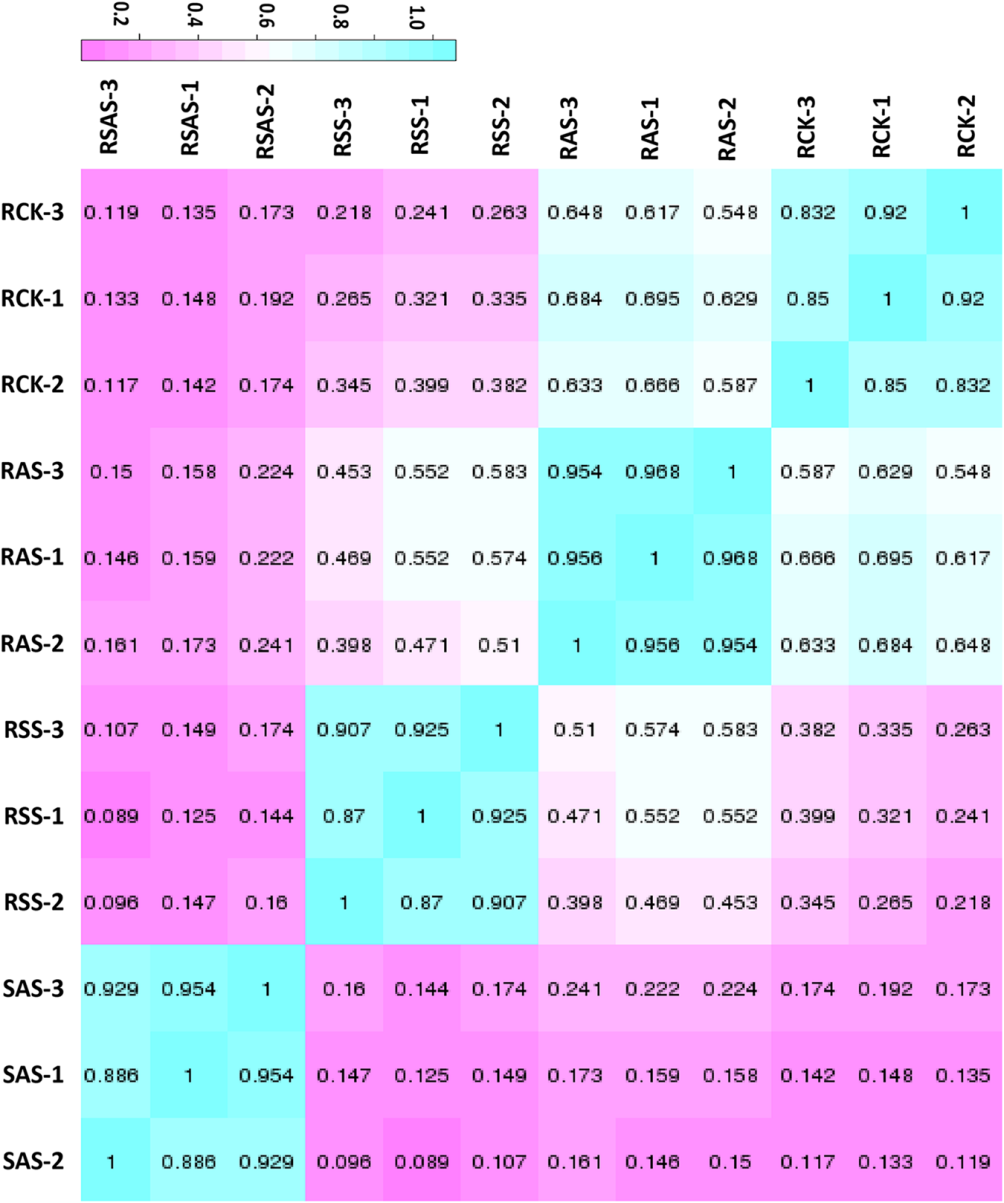
Heatmap of the correlation of the expression level among root samples. RCK: Root, Control group; RSS: Root, 100 mM NaCl; RAS: Root, 0.125 mM NaOH; RSAS: Root, 50 mM Na_2_CO_3_. Numbers in the box refer to Pearson’s correlation coefficient *r*. The colors of box represent the degree of correlation; blue represents a high degree of correlation and pink represents a low degree of correlation.

The aligned sequences were assembled with Cufflinks^21^, which was guided by a annotation genomes of tetraploid species *G*. *hirsutum* from Cottongen^22^. RNA-Seq assays revealed that there were 60,369 unigenes with 95.79% (57,825 unigenes) annotated genes and 9.18% (5,544 unigenes) novel genes (Table 2). For the evaluation of DEGs’ reliability and the filter of abnormal samples, Pearson correlation coefficient (PCC) analysis was conducted. The correlation analysis indicated that under all three salt-alkaline stresses, roots and leaves showed more than 85% similarities except for the genes between RCK-1 and RCK-2 and LSS-1 and LSS-3 (Fig. 2).

**Table 2:** Statistics of functional genes with Cufflinks.

Genes	RSS	RAS	RSAS	LSS	AS	LSAS	Total
All gene	21754	13429	59143	11569	8657	49257	60369
Annotation gene	18436	10122	55903	8129	7672	46078	54825
Novel gene	3318	3307	3240	3440	985	3179	5544

RSS: Root, 100 mM NaCl; RAS: Root, 0.125 mM NaOH; RSAS: Root, 50 mM Na_2_CO_3_. LSS: Leaf, 100 mM NaCl; LAS: Leaf, 0.125 mM NaOH; LSAS:Leaf, 50 mM Na_2_CO_3_.

The gene expression profile under NaOH stress was almost the same as the situation of control group in roots. The gene expression profile under NaCl and Na_2_CO_3_ stresses, however, were different. This result demonstrated that the damage caused by NaOH stress was the slightest one. It also showed the damage caused by Na_2_CO_3_ stress was the most serious situation. The expression correlation analysis showed the similar results in leaves and roots (Fig. S2).

GO (Gene Ontology), KEGG (Kyoto Encyclopedia of Genes and Genomes), NR (RefSeq non-redundant proteins), Swiss-Prot, COG (Cluster of Orthologous Groups), KOG (euKaryotic Ortholog Groups), Pfam and eggNOG (evolutionary genealogy of genes: Non-supervised Orthologous Groups) annotation of the novel genes were conducted. Totally, 4,202 and 4,014 novel genes were annotated in roots and leaves, respectively. We found that genes in roots were enriched in “nucleic acid binding” and “RNA-dependent DNA binding” GO terms. The genes in leaves were found enriched in “nucleic acid binding” and “DNA binding” terms. KEGG pathway analysis indicated that genes were enriched in “carbon metabolism”, “amino acid biosynthesis” and “plant hormone signal transduction” pathways (Fig. S3).

### Differentially expressed genes analysis in leaves and roots under various salt-alkaline stresses and control group

**Figure 3:**
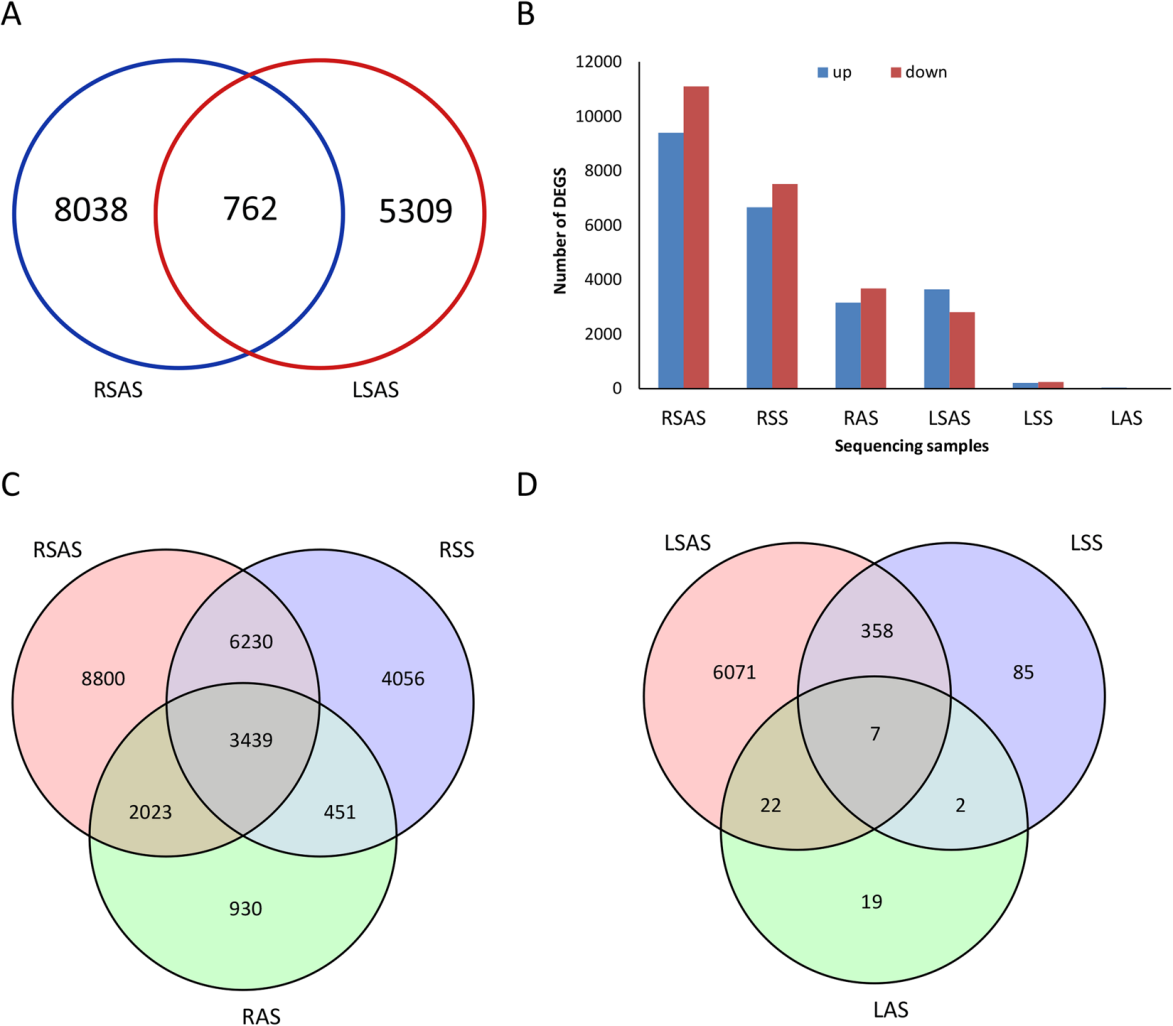
Analysis of differentially expressed genes. RSS: Root, 100 mM NaCl; RAS: Root, 0.125 mM NaOH; RSAS: Root, 50 mM Na_2_CO_3_. LSS: Leaf, 100 mM NaCl; LAS: Leaf, 0.125 mM NaOH; LSAS:Leaf, 50 mM Na_2_CO_3_. (**A**) Specific DEGs in roots and leaves under SAS. (**B**) Number of up-regulated and down-regulated DEGs of each sample. (**C**) Number of DEGs in roots under different stress treatments. (**D**) Number of DEGs in leaves under different stress treatments.

Gene expression levels were estimated by fragments per kilo base of transcript per million fragments mapped (FPKM). Differential expression analysis of treatments and control group was performed using the DESeq. A threshold of Fold Change ≥2 and FDR < 0.01 was used for identifying DEGs. In total, 25,929 DEGs (NaCl: 14,176; NaOH: 6,843; Na_2_CO_3_: 20,492) and 6,564 DEGs (NaCl: 452,NaOH: 50, Na_2_CO_3_: 6,458) were obtained in roots and leaves, respectively. DEGs numbers under different stresses in roots and leaves were presented as Na_2_CO_3_ > NaCl > NaOH stress, which illustrated that Na_2_CO_3_ stress induced a significant biological response in the plant as compare to NaCl stress and NaOH stress. In response to salt-alkaline stresses in roots and leaves, majority of genes got down-regulated in roots and up-regulated in leaves (Fig. 3B). These results indicated their tissue-specificity expression in response to stress^23^.

**Figure 4:**
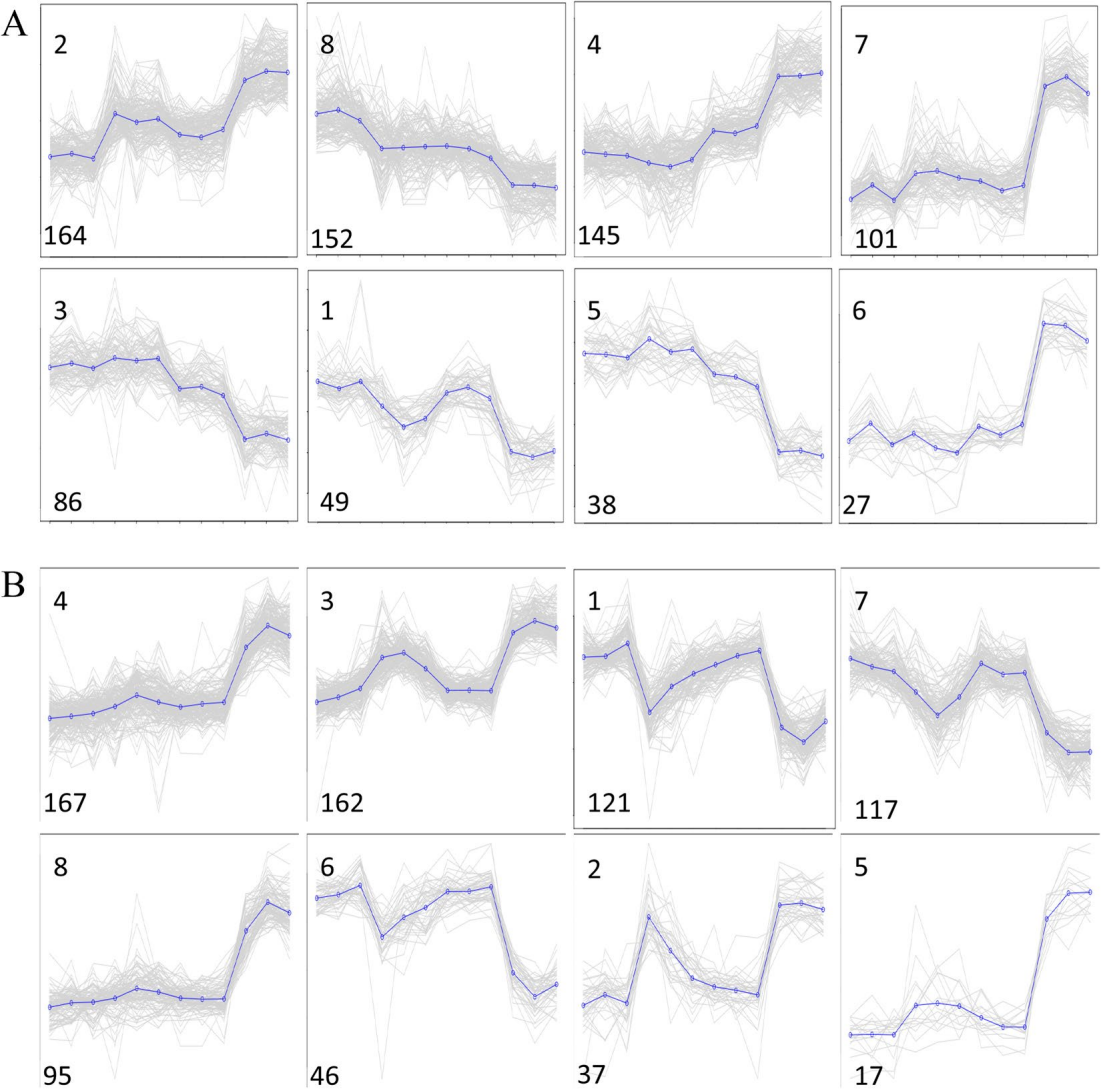
Line graph for the cluster expression of 762 specific DEGs with Na_2_CO_3_ tolerance. (**A**) DEGs in roots. (**B**) DEGs in leaves. The X-axis shows the different treatments(from left to right: CK:normal condition; SS: 100 mM NaCl; AS: 0.125 mM NaOH; SAS: 50 mM Na_2_CO_3_), and the Y-axis shows the standardized FPKM. The number on the bottom right side of cluster panel is cluster number. The number on the bottom left side of cluster panel is genes number of each cluster. Black lines represent the average value of the relative expression level of all genes included in the cluster under different experimental stresses.

**Figure 5:**
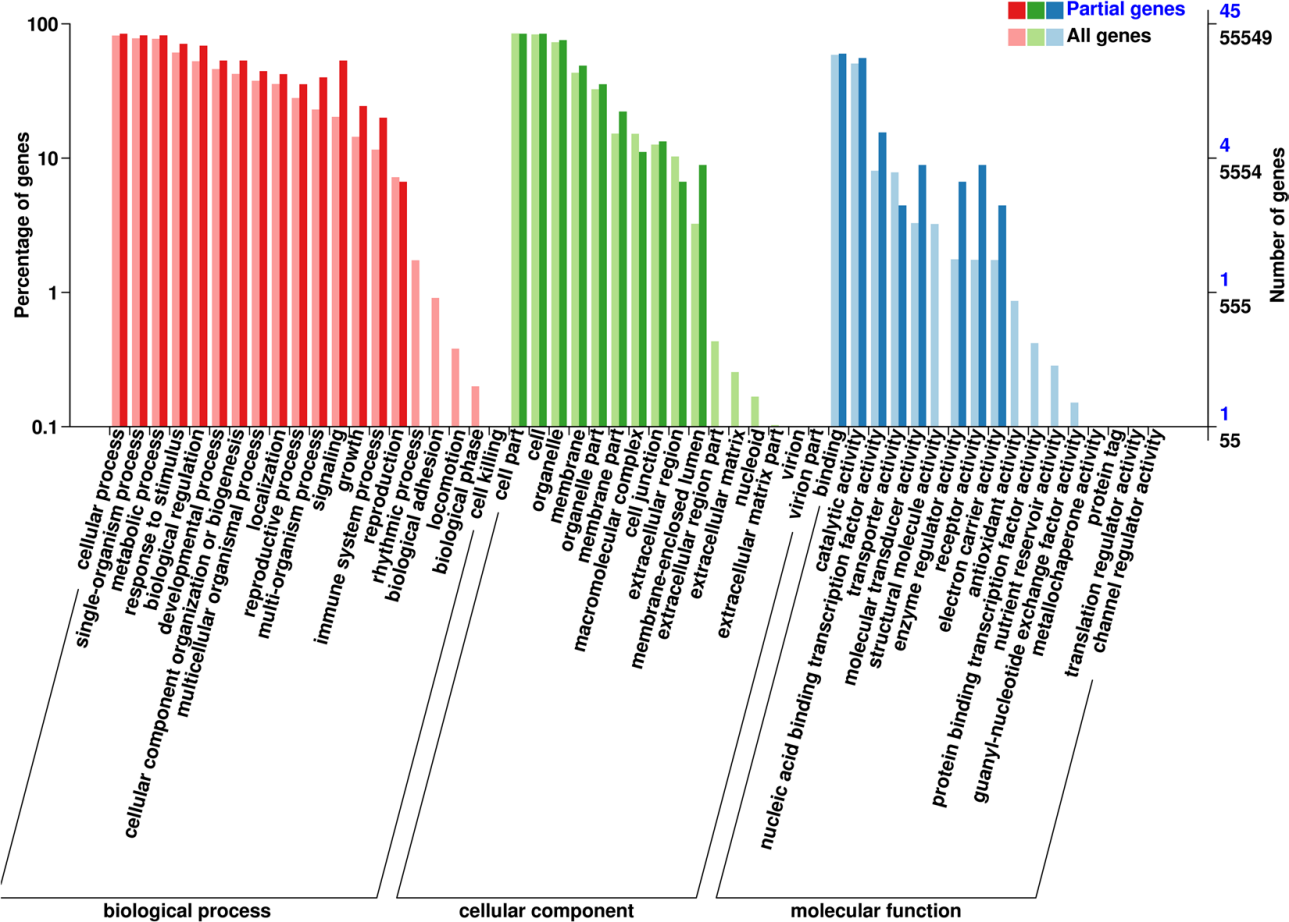
GO classification of cluster4 genes commonly in roots and leaves. X-axis represents the GO terms names: red pillars represent biological process, green pillars represent cellar component, and blue pillars represent molecular function. The deeper colours represent cluster4 genes, the lighter colours represent all genes. The left Y-axis represents percentage of genes, right Y-axis represents number of genes.

Approximately 8,800 (42.94%) of the total DEGs under Na_2_CO_3_ stress were found as root specific and 6,071 (94.00%) were leaf specific DEGs. Furthermore, 4,056 (28.59%) of the total DEGs under NaCl stress were root specific, while 85 (18.8%) DEGs were leaf specific. Moreover, 930 (13.59%) of the total DEGs under NaOH stress were root specific genes and 19 (38.00%) were leaf specific DEGs (Fig. 3C,D). Under Na_2_CO_3_ stress, 762 DEGs were commonly identified in roots and leaves (Fig. 3A). To study the expression profiles of these 762 genes under different salt-alkaline stresses, the FPKM of genes was normalized, and K-means cluster analysis with a normalized FPKM was used. These genes were divided into eight clusters. The genes in each cluster had the same expression profile (Fig. 4). Cluster 2 was the largest one in roots (164 genes, 21.52% of 762 genes), followed by cluster 8 (152 genes, 19.94%), cluster 4 (145 genes, 19.02%) and cluster 7 (101 genes, 13.25%). In leaves, cluster 4 was the largest profile (167 genes, 21.91% of 762 genes), followed by cluster 3 (162 genes, 21.26%), cluster 1 (121 genes, 15.88%) and cluster 7 (117 genes, 15.35%). The similar expression profile in roots and leaves was cluster 4, in which DEGs was up-regulated under Na_2_CO_3_ stress and unchanged under NaCl and NaOH stress. We mapped common cluster 4 genes within roots and leaves (Table S2) in the GO database. Results are presented in Fig. 5.

### GO enrichment analysis of differentially expressed genes

To further investigate the function of DEGs, we mapped all of DEGs to the GO database^24^. 5,769 (87.89%) and 20,508 (79.09%) DEGs in leaves and roots were annotated, respectively. “Response to stimulus” (GO:0050896) was enriched in both leaves and roots after the results were classified. The enriched DEGs were different among the salt-alkaline stresses (Table 3), which were consistent with the phenotypic changes under different stresses. Under NaCl and NaOH stress, more genes related to “response to stimulus” were identified in roots than in leaves (Table 3), while more genes were recognized in roots as compared to those in leaves under Na_2_CO_3_ stress. The results implied that a more complicated physiological process occurred in roots than in leaves when the cotton plants were damaged by both NaCl and NaOH stresses. This was in contrast to the situation when seedlings were under Na_2_CO_3_ stress.

**Table 3:** Analysis of GO enrichment terms in response to stimulus.

Term	ID	RSS	LSS	RAS	LAS	RSAS	LSAS
divalent metal ion transport	GO:0070838	0.106702 29/409	0.440725 1/409	0.323192 7/409	0	0.974428 38/409	0 86/409
hyperosmotic salinity response	GO:0042538	0.000942 154/1857	0.49109 3/1857	0.468253 26/1857	0	0.999992 170/1857	0 274/1857
cellular cation homeostasis	GO:0030003	0.075488 42/601	0.208845 2/601	0.07003 13/601	0	0.996858 53/601	0 110/601
protein serine/threonine phosphatase activity	GO:0004722	0.003649 50/447	0	0.854654 4/447	0	0.141608 64/447	0.369568 44/447
response to osmotic stress	GO:0006970	0.052443 72/787	0.02548 4/787	0.189197 14/787	0	0.611997 89/787	0.0241 89/787
		347	10	64	0	414	603
total	1084						

RSS: Root, 100 mM NaCl; RAS: Root, 0.125 mM NaOH; RSAS: Root, 50 mM Na_2_CO_3_. LSS: Leaf, 100 mM NaCl; LAS: Leaf, 0.125 mM NaOH; LSAS:Leaf, 50 mM Na_2_CO_3_. Each box shows the genes numbers and the p-value. The pair of numerals in the left represents number of genes in input list. The pair of numerals in the right represents number of genes in the TM-1 database.

**Figure 6:**
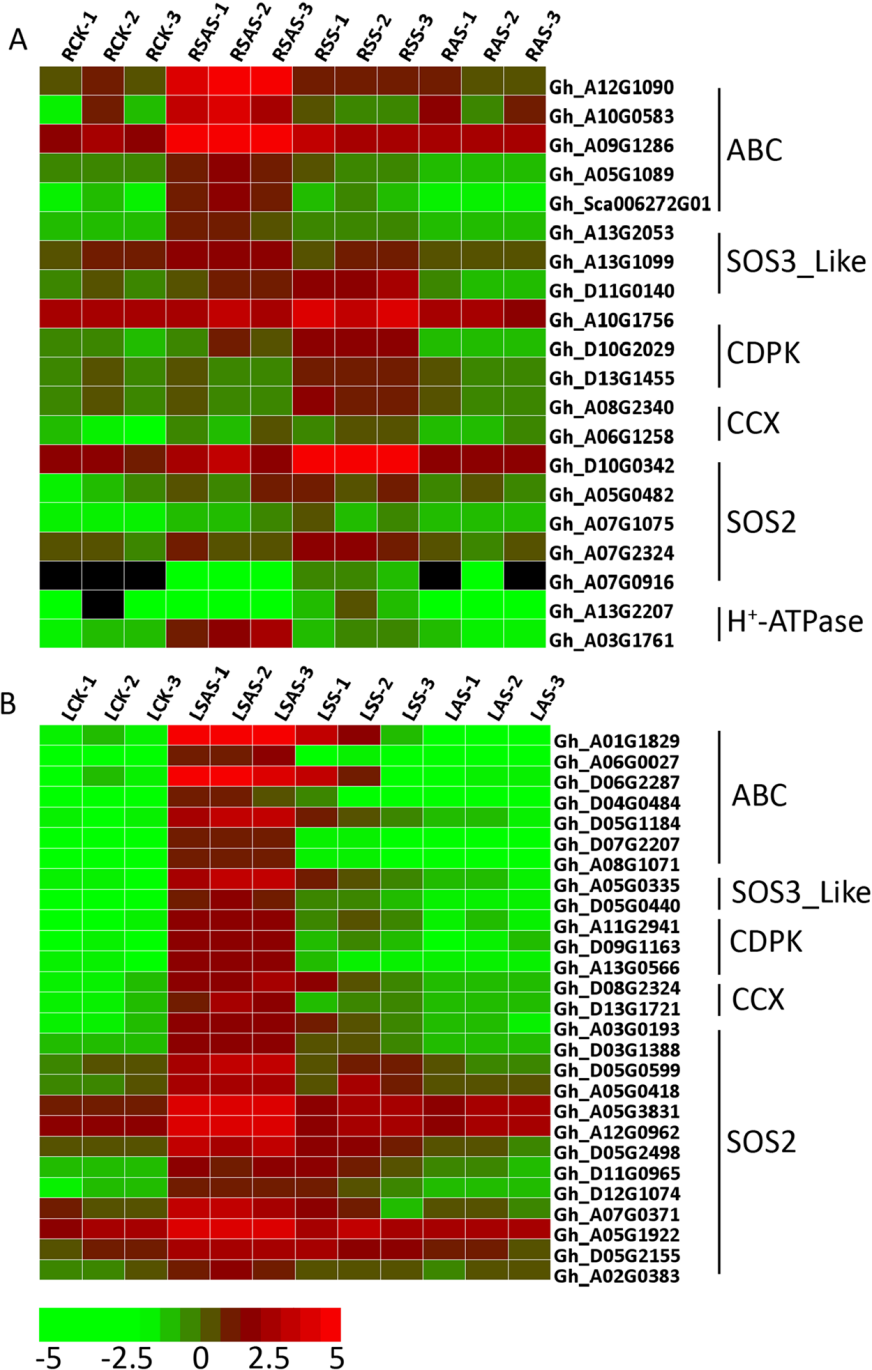
Heatmap for the co-expression of genes clusters related to ionic homeostasis in roots and leaves under different stresses. RCK: Root, Control group; RSS: Root, 100 mM NaCl; RAS: Root, 0.125 mM NaOH; RSAS: Root, 50 mM Na_2_CO_3_, LCK: Leaf, Control group; LSS: Leaf, 100 mM NaCl; LAS: Leaf, 0.125 mM NaOH; LSAS: Leaf, 50 mM Na_2_CO_3_. (**A**) Heat map log_2_ FC (Fold Change) value of the expression level cluster of roots under different stresses. (**B**) Heat map log_2_ FC (Fold Change) value of the expression level cluster of leaves under different stresses. Red = high expression level of genes, and Green = low expression level of genes.

Functional enrichment of the annotated genes was conducted using topGO^25^, and the results of the enrichment were sorted by p-value numbers. Then the first 20 GO terms with the smallest p-values were chosen. GO enrichment analysis revealed an enrichment of genes involved in plant responses to salt stress, ionic homeostasis, organic substance, hormone signal pathways and osmotic stress in leaves and roots under salt-alkaline stresses (Fig. S4). The results revealed that “divalent metal ion transport”, “hyperosmotic salinity response” and “cellular cation homeostasis” genes had higher expression levels under Na_2_CO_3_ stress. “Hyperosmotic salinity response”, “response to osmotic stress” and “protein serine/threonine phosphatase activity” genes reached a higher expression level in NaCl stress, while these genes were non-significantly enriched under Na_2_CO_3_ stress. These genes are related to osmotic adjustment and maintenance of intracellular ionic homeostasis, playing vital roles in plant tolerance to salt stress^26^. According to statistics, DEGs under SS, AS and SAS appeared as following: Na_2_CO_3_ > NaCl > NaOH stress (Table S3). Genes related to ion absorption and compartmentalization, such as SOS2, SOS3-Like, CCX, CDPK and ABC transporters, were detected from these genes^27–31^. These were up-regulated under Na_2_CO_3_ and NaCl stress, and were down-regulated under NaOH stress (Fig. 6).

### KEGG metabolic pathways annotation of sugar metabolism in responses to different alkaline stresses

Annotation analysis of the Kyoto Encyclopedia of Genes and Genomes (KEGG) pathways^32^ of DEGs contributed to the decoding of gene functions. A total of 1,392 (21.21%) and 5,510 (21.25%) DEGs were annotated in roots and leaves, respectively. The KEGG enrichment pathways (Fig. S5) indicated that the specific DEGs, found under three salt-alkaline stresses in roots and leaves, were widely enriched in the pathways of plant hormone signal transduction^33^, photosynthesis, peroxidase^34,35^ and glutathione metabolism^36^. According to previous studies, these pathways are related to abiotic stresses tolerance.

**Figure 7:**
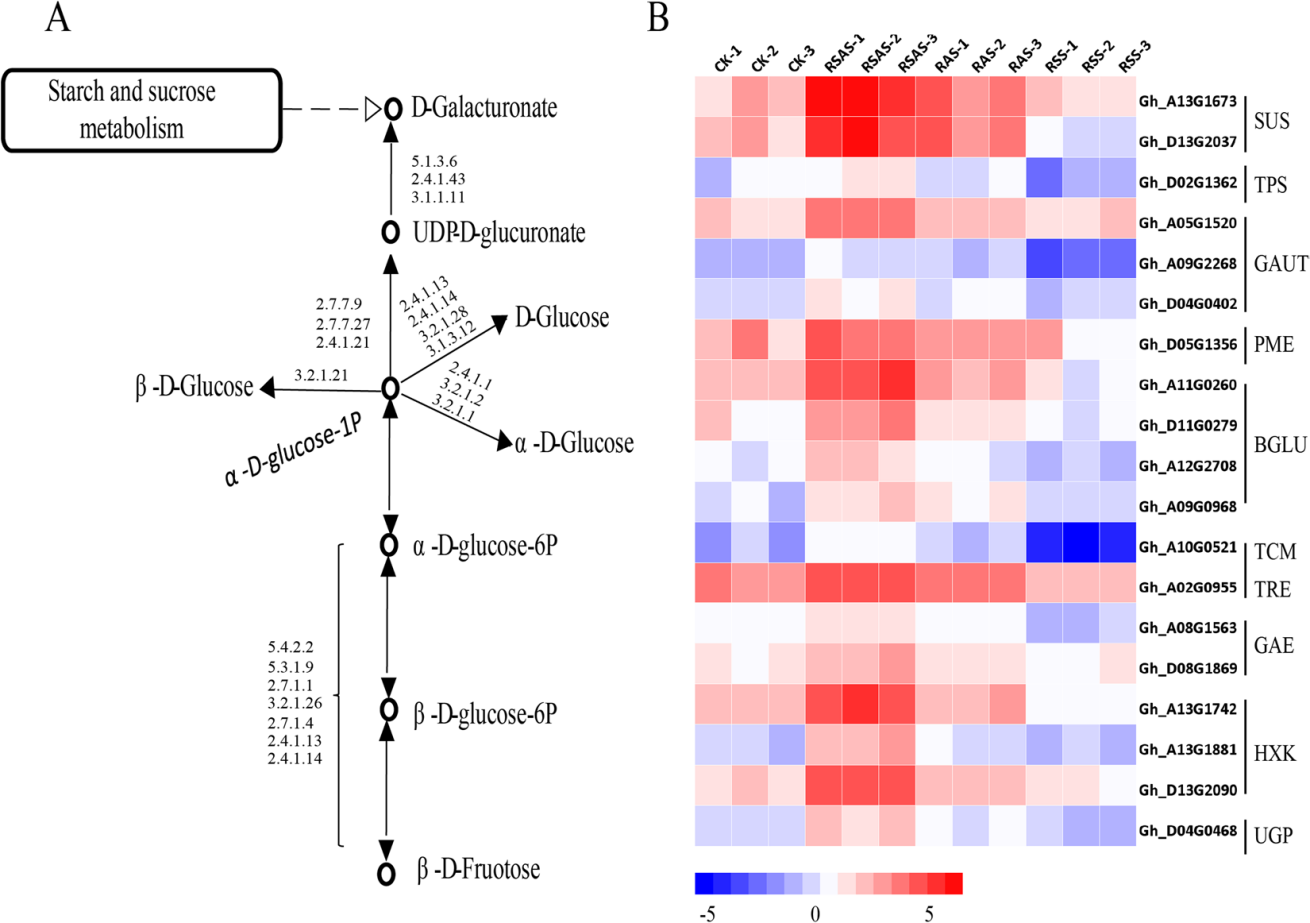
Analysis of differential genes in pathways of starch and sucrose Metabolism. RCK: Root, Control group; RSS: Root, 100 mM NaCl; RAS: Root, 0.125 mM NaOH; RSAS: Root, 50 mM Na_2_CO_3_. (**A**) A schematic diagram of sugar metabolism. *Digits* represents regulatory enzyme for specific process. (**B**) Up-regulated DEGs under Na_2_CO_3_ and NaOH treatments and down-regulated DEGs under NaCl treatment.

DEGs were significantly enriched in the pathway “starch and sucrose metabolism” (ko00500) under NaOH and Na_2_CO_3_ stresses, but not significantly enriched under NaCl stress. The sucrose content of leaves increased significantly when *lupin* was under NaCl stress^37,38^. It has reported that exogenous sugar affected the hormone signal transduction, key enzyme metabolism and sucrose metabolism of grape seedlings^39^. Among DEGs enriched in starch and sucrose metabolism (Figs 7A and S6), we found some genes that had up-regulated under NaOH and Na_2_CO_3_ stress and down-regulated under NaCl stress in roots (Fig. 7B). The TPS1-TPS2 (trehalose-6-phosphate synthase) lines displayed a significant increase in drought, freezing, salt and heat tolerance^40^.

### Transcription factors analysis and annotation

**Figure 8:**
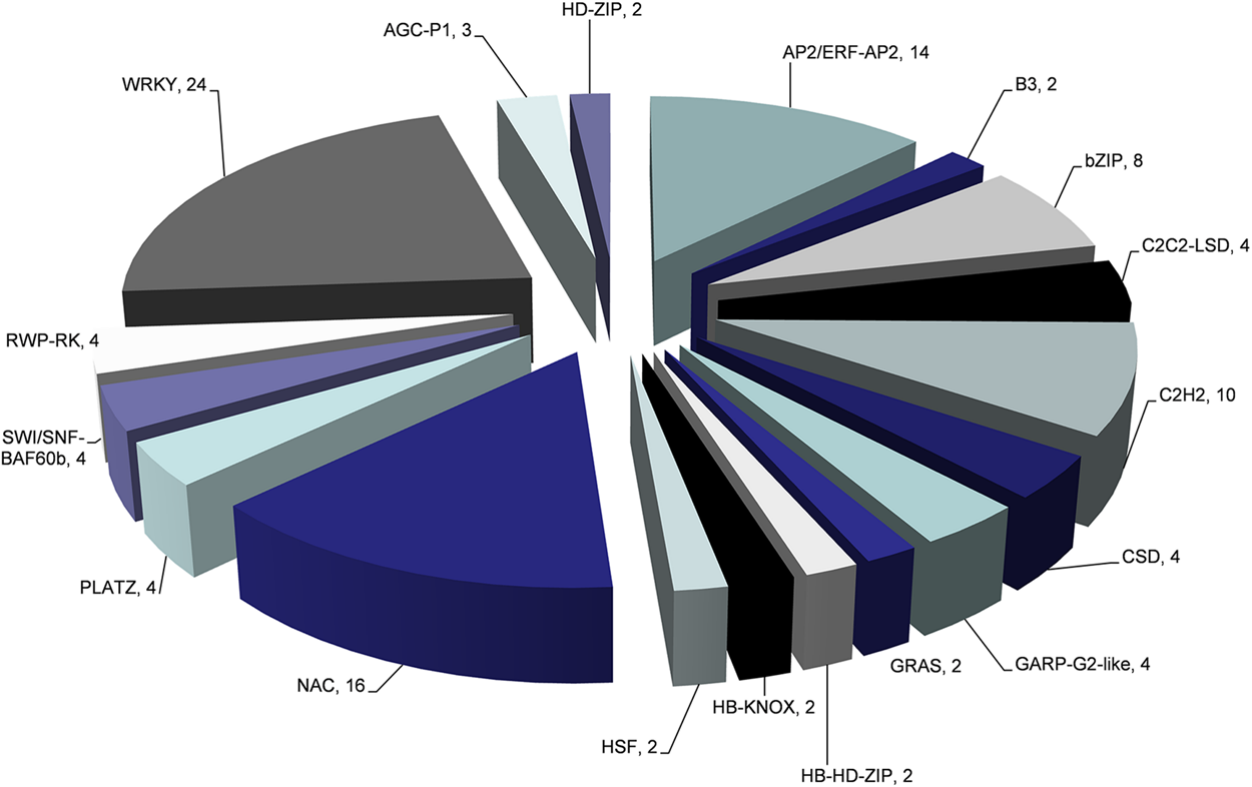
Annotation of transcription factors of specific DEGs tolerance to Na_2_CO_3_ stress.

Many studies have reported that transcription factors (TFs) play an important role in stress tolerance^41^. Transcription factor annotation was performed among 762 specific DEGs (Fig. 3A). These TFs were classified into 18 families and three protein kinases families. Except for bZIP (basic region/leucine zipper)^42^, NAC (NAM/no apical meristem, ATAF/Arabidopsis transcription activation factor, and CUC/cup-shaped cotyledon)^43^, MYB (v-myb avian myeloblastosis viral oncogene homolog)^44^ and ERF (ethylene response elements)^45^ families were related to salt-alkaline tolerance, C2H2 (Cys2/His2) transcription factor family was also enriched (Fig. 8). C2H2, a zinc finger protein, is related to the osmotic stress tolerance of *Arabidopsis thaliana*^46–48^.

### Validation of RNA-Seq data by quantitative real-time PCR

To verify the reliability of sequencing, quantitative real-time PCR (qRT-PCR) was performed using the same RNA samples that were previously used for RNA-seq. A total of 20 genes were randomly selected for qRT-PCR, including 10 up-regulated genes and 10 down-regulated genes. Linear correlation analysis of the data of these two groups was performed. The fold change (FC) of genes between salt-alkaline-stressed tissues and controlled treatments using qRT-PCR were compared to those ones using RNA-Seq. The correlation coefficients between qRT-PCR and RNA-seq were significant in the roots and leaves under different salt-alkaline stresses (Fig. S7).

## Discussion

### Synergistic enhancing effect of salt stress and alkaline stress

Salt-alkaline situation often results in osmotic stress to plants firstly, following by ionic toxicity and consequently oxidative stress, which leads to an increase in intracellular active oxygen^6^. Numerous studies on molecular mechanisms of cotton tolerance to salt stress have been conducted by mainly focusing on salt tolerance, but few have investigated the difference between salt tolerance and alkaline tolerance. In comparison with the situation under NaCl and NaOH stresses^3,7^, maize seedlings suffered more changes under Na_2_CO_3_ stress. In this study, significant phenotypic differences were observed in roots and leaves under Na_2_CO_3_ stress.

RNA-seq analysis of roots and leaves under NaCl, NaOH and Na_2_CO_3_ stresses was performed to investigate different molecular mechanisms under salt and alkaline stress. The expression profiles induced by Na_2_CO_3_ and NaCl in *Puccinellia tenuiflora*^3^ and maize^7^ were relatively pronounced, while those induced by NaOH were relatively unchanged. Results indicated that NaOH stress have triggered few biological responses, probably related to the neutralization of pH by organic acid, such as ascorbic acid accumulation^4^, alleviating serious damage. Significant biological responses occur under NaCl stress, because plants cannot eliminate ions immediately and may constantly suffer from osmotic stress and ionic toxicity^6,49^. More biological responses may occur in plants if there is both high pH and salt stress under Na_2_CO_3_ stress. In our study, the DEGs numbers are presented as: Na_2_CO_3_ > NaCl > NaOH stress which showed in Fig. 3. It indicates that there is a complicated and synergistic effect between NaCl and NaOH stresses. It also explains that NaCl and NaOH stresses together exhibit significant damages to plants.

### Ion homeostasis in response to Na^+^ stress

Plant under salt-alkaline stress may first generate a series of substances for osmotic adjustment and then regulate relevant genes expression to maintain ionic homeostasis and balance the cellular osmotic potential^6,49^. According to GO function enrichment of DEGs, we found that the GO enrichment terms “hyperosmotic salinity response”, “response to osmotic stress”, “protein serine/threonine phosphatase activity”, “divalent metal ion transport”, and “cellular cation homeostasis” genes, which are related to osmotic adjustment and maintenance of intracellular ion homeostasis, were enriched under NaCl and Na_2_CO_3_ stresses, but not under NaOH stress^27,30^. Hence, we paid close attention to these genes related to osmotic adjustmentand ion homeostasis. As shown in Fig. S4, there may be a special mechanism of Na^+^ stress tolerance.

The ABC (ATP-binding cassette) transporters use the energy produced by hydrolysis of ATP for the transportation of micromolecules and play a vital role in ion homeostasis^50^. In our study, several ABC transporter genes were up-regulated under NaCl and Na_2_CO_3_ stresses and down-regulated under NaOH stress. We found that the *ABCB*21 (Gh_A12G1090), *ABCG*36 (Gh_A10G0583, Gh_A05G1089, and Gh_Sca006272G01), and *ABC*2 (Gh_A09G1286) genes in the roots of *Gossypium hirsutum* were up-regulated under Na^+^ stress. Kamimoto *et al*. demonstrated that *Arabidopsis* ABCB21 imported/exported auxin^51^. Auxin (IAA) regulated NAC transcription factors of *NTM*2 expression under salt stress^52^. In our study, we found that the NAC family was enriched under Na_2_CO_3_ stress. Kim *et al*. found that *AtPDR*8 (*ABCG*36) was an efflux pump of Cd^2+^ ^53^. *AtPDR*12 mediates the uptake of ABA (abscisic acid) in *Arabidopsis*^54^. ABA stimulates calcium-dependent protein kinases (CDPKs) and serine/threonine phosphatase activity (SOS2).The level of SOS2 and SOS3-Like (also known as CBLs) proteins were both up-regulated in roots and leaves under Na^+^ stress. The protein SOS2 can regulate Na^+^/K^+^ transport proteins and CBLs worked as a calcium sensor in plant salt tolerance^55^. *cpk* mutation is salt-insensitive^56^. CBLs and CDPKs can alter the transcriptional profile, such as the transcription factors (MYB, WRKY, and bZIP), which are regulated and expression of the downstream genes is changed^6^. MYBs regulated the genes of the anthocyanin pathway in Rosaceae^57^. Kim et al. introduced a double knockout mutant of bZIP17 and bZIP28 to analyze the function of bZIP17^58^. They found that mutant plants exhibited multiple developmental defects including scarce root elongation. These TFs were significantly up-regulated under Na^+^ stress in roots and leaves. We also found that CCX-related genes were up-regulated. Chen *et al*. speculated that *AtCCX*1 is vital for Na^+^ resistance and serves as a Na^+^/K^+^ exchanger in vacuoles.

These results indicate that ionic homeostasis may not be affected by the high pH induced by NaOH stress. However, high pH may increase osmotic stress together with Na^+^ and produce a synergistic enhancing effect of NaCl and NaOH stresses. The reason is that Na^+^ easily affects the dynamic equilibrium of the cytomembrane under environment with high pH^4,59,60^. The thorough mechanism of the synergistic enhancing effect needs further study.

### Oxygen deficiency in response to high pH

For further detection of the differences between salt and alkaline stresses, KEGG pathways enrichment analysis of DEGs was performed. The results demonstrate that under NaOH and Na_2_CO_3_ stresses, DEGs were found significantly enriched in “starch and sucrose metabolism” pathways, but non-significantly under NaCl stress. It has been noted in results that genes enriched with this pathway may have some relationship with high pH. It has been reported in previous studies that the cytosolic pH of maize roots decreases from 7.5 to 6.5 under oxygen deprivation^61^. Annabelle *et al*. found that the transcription level of *Sus2* (sucrose synthase) was ABA-dependent and specifically induced by O_2_ deficiency^62^. We found that genes encoding *Sus* were up-regulated under high pH stress (under both NaOH and Na_2_CO_3_ stresses, but not NaCl stress). Hence, we speculated that high pH results insufficient oxygen level in plant cells, followed by induction of *Sus* genes and increased transformation of glucose into sucrose β-glucosidase (BGLU) was accumulated in the ER (endoplasmic reticulum) body, which plays a vital role in the defense system of plants^63^. β-glucosidase is a substrate of glucose production, and *BGLU* genes were up-regulated. Plants use HXK (hexokinase) as a glucose sensor in response to a changing environment^63^. Mu *et al*. showed that trehalose-6-phosphate synthase (TPS) genes are stress-related in cotton^64^. Nelson *et al*. found that *AtTPS*1 is probably a member of the *HXK1*-dependent Glc-signaling pathway^65^, and that O_2_ deficiency is regulated by the expression of *Ugp*^66^. Studies have reported that the use of exogenous sugar may affect hormone signal transduction, key enzyme metabolism and sucrose metabolism of grape seedlings^39^. Genes enriched in “starch and sucrose metabolism” pathways are up-regulated under NaOH and Na_2_CO_3_ stress and down-regulated under NaCl stress. These genes then participate in plant hormone signal transduction and affect the activities of some key enzymes (such as ROS, ATPase and *RuBisCO*). Galacturonosyltransferases (GalATs/GAUT) are required for the synthesis of pectin^67^. Pectin methylesterase (PME) catalyzes pectin deesterification, releasing acid pectin and methanol, which cause cell wall changes^68^. The Subcellular locations of GAUT, PME and UDP-D-glucuronate 4-epimerase (GAE) are Golgi^69^, suggesting that PME, GAUT and GAE were related to synthesis of cell wall.

The expression of these genes indicates that high pH firstly causes oxygen deprivation stress to cotton plants. Lacking of oxygen leads to anaerobic respiration of plants and produce ethylene which causes roots rotten^70^. In addition, there were nigrities in roots under NaOH and Na_2_CO_3_ stresses and in leaves under Na_2_CO_3_ stress. Hence, we speculated the phenotypic changes under high pH were related to the oxygen deprivation signal pathway. Consequently, glucose synthesis and decomposition, TPS synthesis and other sugar metabolisms are involved in defending against oxygen deprivation. What is more, genes related to the synthesis of pectin were up-related. Pectin is an important cell wall polysaccharide that allows primary cell wall extension and plant growth^68^. We inferred that high pH induce genes that recoded proteins of cell wall synthesis (PME, GAUT and GAE) were up-regulated to strengthen cell wall and defend high pH damage.

## Conclusion

**Figure 9:**
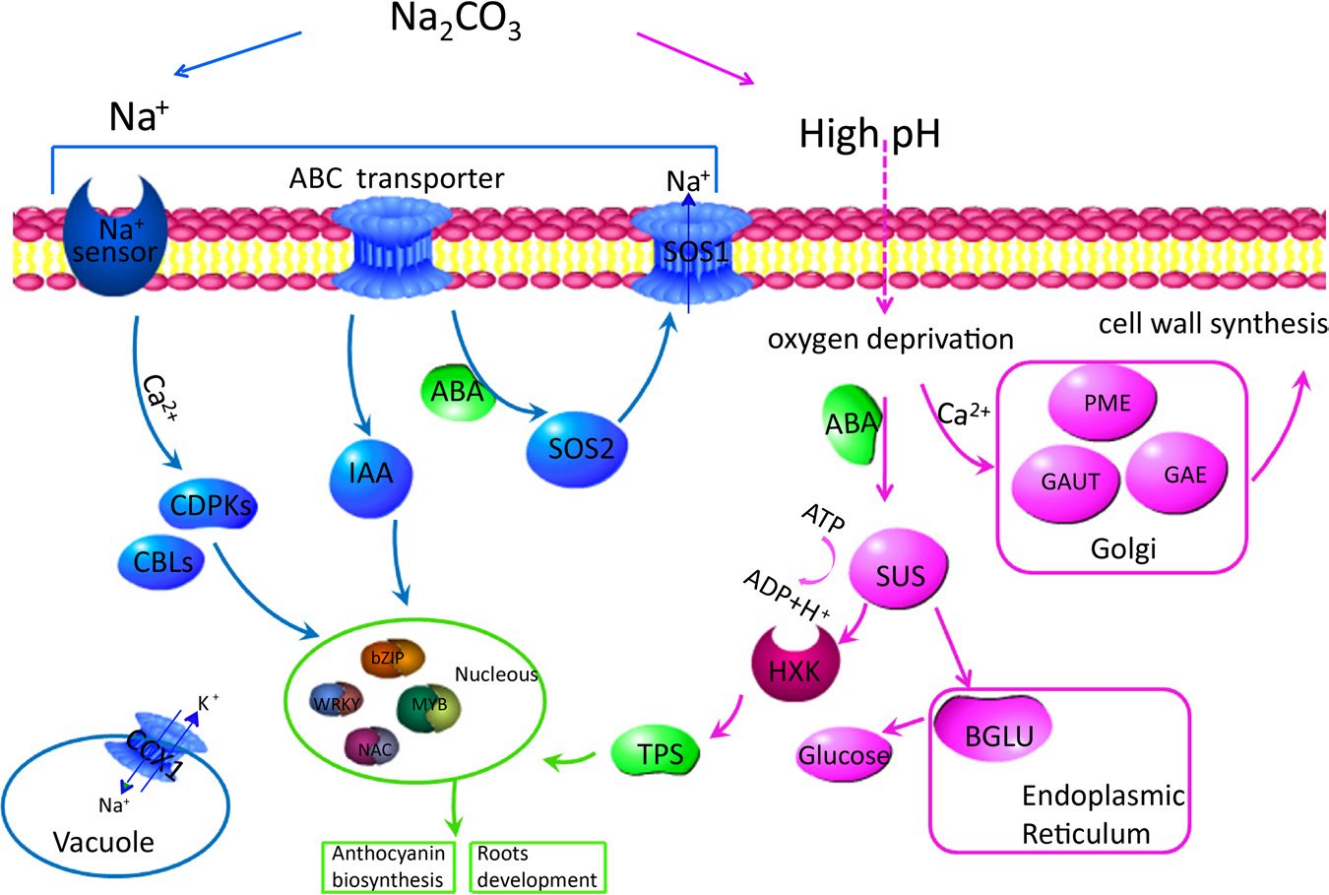
Model of the regulatory networks in response to Na^+^ stress and high pH. The left blue part of the networks is related to Na^+^ stress, the right red part of the networks is related to high pH. The crossed green part is related to both Na^+^ stress and high pH.

This paper described the possible mechanism response to Na_2_CO_3_ tolerance in cotton. In general, most of the genes and proteins related to Na^+^ stress (treated with NaCl) and high pH (treated with NaOH) are also involved in the pathways against Na_2_CO_3_ stress (Fig. 9). We speculate that cotton may regulate the metabolism of starch and sucrose due to the toxicity of high pH, which results in the specifical expression of some genes under Na_2_CO_3_ and NaOH stresses. High pH leads to oxygen deprivation stress, which causes cotton organs nigrities and rottenness. In addition, high pH also increases the synthesis of pectin-related enzymes, which strengthens cell wall to defense damage of high pH. In the process of the hydrolysis of ATPase, extra H^+^ produced help to neutralize OH^−^ within the cytoplasm.

Besides, genes and proteins related with ion homeostasis under Na^+^ stress were also found in our study, such as protein kinases, transcription and transporters (Fig. 9). And these genes and proteins have been reported in previous studies. MYBs that regulate genes of the anthocyanin pathway were up-regulated under Na_2_CO_3_ stress, which always cause leaves to turn red or orange in apple^71^. bZIPs play a important role in roots development under salt stress^58^.

The mechanism of salt and alkaline tolerance in cotton are complicated. Further study is still needed, even though several genes have been transported into cotton and other plants. Here our study provides some candidate genes particularly responsing to high pH and Na^+^ stresses. For instance, Hexokinase (HXK) acted as a sugar sensor in eukaryotic cells^72^, being found to be up-regulated under high pH stress, which indicates that genes encoding HXK may be related with high pH stress.

## Materials and Methods

### Plant materials and salt-alkaline stresses conditions

*G*. *hirsutum* cultivars Zhong9807 was used for this study. Seeds were sown in sand soil pots.The sand was washed cleanly and sterilized at 121 °C for 8 h. Four seedlings in each pot were cultivated in a 28 °C/14 h light and 25 °C/10 h dark cycle with a light intensity of 150 μmol m^−2^ s^−1^and 75% relative humidity for approximately 30 days. Seedlings containing three true leaves and one heart-shaped leaf were washed out carefully and transplanted into conical flasks containing 0, 25, 50, 75, and 100 mM Na_2_CO_3_ solution for 0, 6, 12, and 24 h to observe phenotypic changes. Then, 50 mM Na_2_CO_3_ over 12 h was chosen as the applicable stress concentration and time. In addition, for salinity stress treatment, seedlings were transferred into ddH_2_O supplemented with 100 mM NaCl (pH = 7.0). For alkaline stress treatments, seedlings were transplanted into ddH_2_O supplemented with 0.125 mM NaOH (pH = 11.32) and 50 mM Na_2_CO_3_ (pH = 11.32). Seedlings transplanted into normal ddH_2_O were used as controls. After exposure for 12 h, antepenultimate leaf and whole root samples were collected. Each sample was tested three time. Samples were frozen in liquid nitrogen and stored at −80 °C for physiological measurement and transcriptome analysis.

### Measurement of relative water content and chlorophyll content

The relative water content (RWC) of roots and leaves was calculated following the method described previously^73^. The total chlorophyll content of leaves was measured spectrophotometrically using the method of Arnon^74^, and variance analysis proceeded as described by Anders^19^.

### cDNA libraries preparation and RNA-seq

Total RNA of roots and leaves was extracted. RNA degradation and contamination was monitored on 1% agarose gels. RNA purity was checked using the NanoPhotometer spectrophotometer (IMPLEN, CA, USA). RNA concentration was measured using Qubit RNA Assay Kit in Qubit 2.0 Flurometer (Life Technologies, CA, USA). RNA integrity was assessed using the RNA Nano 6000 Assay Kit of the Agilent Bioanalyzer 2100 system (Agilent Technologies, CA, USA). Qualified RNA samples were randomly digested with fragmentation buffer. Library preparation for RNA-Seq was performed using the Employed NEB Next Ultra TM RNA Library Prep Kit (NEB, USA). Library quality was assessed on the Agilent Bioanalyzer 2100 system. The clustering of the index-coded samples was performed on a cBot Cluster Generation System using TruSeq PE Cluster Kit v4-cBot-HS (Illumia) according to the manufacturer’s instructions. After cluster generation, the library preparations were sequenced on an Illumina Hiseq. 2500 platform and paired-end reads were generated.

### Quality control, alignment and differential expression genes analysis

Raw data in the fastq format was first processed through in-house perl scripts. The adaptor sequences and low-quality sequence reads were removed from the data sets. Raw sequences were transformed into clean reads after data processing. These clean reads were mapped to the *G*.*hirsutum* genome using TopHat2 tool^20^. These mapped reads were spliced using Cufflinks software^21^ based on the reference genome sequence. Quantification of the gene expression levels were estimated as fragments per kilobase of transcript per million fragments mapped (FKPM)^75^. Differential expression analysis of two groups was performed using the DESeq R package (1.10.1). DESeq provides statistical routines for determining differential expression in digital gene expression data using a model based on the negative binomial distribution. The resulting P values were adjusted using Benjamini and Hochberg’s approach for controlling the false discovery rate. Genes with an adjusted P-value < 0.05 were found using DESeq^76^ and were assigned as differentially expressed. Consequently, DEGs were obtained of three biological conditions. Fold Change ≥2 and FDR < 0.01 were taken as the thresholds for determining whether a gene had differential expression.

### Gene ontology and gene pathway enrichment analysis

Gene Ontology (GO) enrichment analysis of the DEGs was implemented using the GOseq R packages based on the Wallenius non-central hyper-geometric distribution, which can adjust for gene-length bias in DEGs^24^. KEGG^32^ is a database resource for understanding high-level functions and utilities of the biological systems, such as the cell, organism and ecosystem, from molecular-level information, especially large-scale molecular datasets generated by genome sequencing and other high-throughput experimental technologies (http://www.genome.jp/kegg/). We used KOBAS^77^ software to test the statistical enrichment of differential expression of genes in KEGG pathways.

### qRT-PCR verification of RNA-seq data

qRT-PCR was carried out using the same samples. 20 genes were chosen randomly (Table S4), including 10 up-regulated and 10 down-regulated genes from the roots and leaves according to the FPKM. qRT-PCR was performed using the Applied Biosystems^@^7500 Fast instrument and *TransStart* Top Green qPCR SuperMix. Reactions were performed with three technological and biological repetitions: 0.4 μL of each primer (10 μM/μL), 0.4 μL of passive reference Dye and 10 μL of Top Green qPCR Supermix at a final volume of 20 μL. The profile for amplification was as follows: 5 min at 95 °C, followed by 40 cycles amplification of 95 °C for 15 s, then 20 s at 58 °C, and 30 s at 72 °C. The ΔΔCt method was used to calculate the relative fold change for each sample^78^. The *GhHis3* house-keeping gene was used as a control. The correlation coefficients between qRT-PCR and RNA-seq was performed.

## Electronic supplementary material

Supplementary Information
