# Supplementary material for: Transcriptome Analysis of Gossypium hirsutum L. Reveals Different Mechanisms among NaCl, NaOH and Na2CO3 Stress Tolerance
Source: Sci Rep. 2018 Sep 10;8:13527. doi: 10.1038/s41598-018-31668-z (PMC6131252; doi:10.1038/s41598-018-31668-z)
Supplement: Supplementary file 1 — Supplementary Information [file 41598_2018_31668_MOESM1_ESM.docx]

Supplementary Information

**Transcriptome Analysis of *Gossypium hirsutum* L. Reveals Different Mechanisms among NaCl, NaOH and Na_2_CO_3_ Stress Tolerance**

Binglei Zhang^1^, Xiugui Chen^1^, XukeLu^1^, Na Shu^1^, Xiaoge Wang^1^, Xiaomin Yang^1^, Shuai Wang^1^, Junjuan Wang^1^, Lixue Guo^1^, Delong Wang^1^ & Wuwei Ye^1*^

^1^State Key Laboratory of Cotton Biology/Institute of Cotton Research of Chinese Academy of Agricultural Sciences/Key Laboratory for Cotton Genetic Improvement, Anyang 455000, Henan, China

*Author for correspondence. WY: yew158@163.com

Supplementary Information contains:

Supplementary Table S1-S4

Supplementary Figure S1-S7

**Table S1.** Mapping result of RNA-Seq reads

| **Libabry** | **clean reads** | **Map reads** | | **≥Q30（%）** |
| --- | --- | --- | --- | --- |
| RCK-1 | 142,375,744 | 114,044,128 | 88.85% | |
| RCK-2 | 103,844,032 | 82,246,947 | 88.34% | |
| RCK-3 | 118,835,418 | 95,566,228 | 88.88% | |
| RSAS-1 | 52,092,294 | 42,304,057 | 87.74% | |
| RSAS-2 | 57,432,998 | 46,799,856 | 87.67% | |
| RSAS-3 | 49,838,212 | 40,807,804 | 88.25% | |
| RSS-1 | 48,441,964 | 39,421,693 | 89.25% | |
| RSS-2 | 58,078,020 | 46,520,167 | 88.57% | |
| RSS-3 | 57,704,056 | 47,076,132 | 89.57% | |
| RAS-1 | 43,515,188 | 34,977,131 | 89.32% | |
| RAS-2 | 46,740,390 | 38,998,243 | 90.12% | |
| RAS-3 | 43,623,754 | 34,752,098 | 88.77% | |
| LCK-1 | 44,370,416 | 36,260,362 | 87.23% | |
| LCK-2 | 41,482,892 | 33,965,143 | 87.53% | |
| LCK-3 | 59,550,982 | 48,563,473 | 87.83% | |
| LSAS-1 | 45,361,644 | 37,246,330 | 87.49% | |
| LSAS-2 | 58,313,150 | 47,147,869 | 87.02% | |
| LSAS-3 | 59,121,742 | 48,172,095 | 87.28% | |
| LSS-1 | 54,341,600 | 42,511,566 | 87.50% | |
| LSS-2 | 43,811,420 | 36,046,644 | 87.77% | |
| LSS-3 | 49,684,216 | 40,776,177 | 87.69% | |
| LAS-1 | 54,947,978 | 44,858,371 | 89.42% | |
| LAS-2 | 53,221,034 | 43,330,188 | 89.15% | |
| LAS-3 | 49,175,576 | 40,354,967 | 89.54% | |

**Table S2. The common genes of cluster 4 under Na_2_CO_3_ stress in roots and leaves**

| Gene ID | function annotation | Gene ID | function annotation |
| --- | --- | --- | --- |
| Gh_A01G1079 | cholesterol/phospholipid flippase | Gh_D03G0439 | ABC transporter |
| Gh_A02G0535 | hypothetical protein | Gh_D03G0538 | ROOT HAIR DEFECTIVE 3 |
| Gh_A02G0711 | Phosphatase 2C family | Gh_D04G0019 | ABA receptor |
| Gh_A05G1676 | serine/threonine-protein kinase | Gh_D04G0098 | chloroplastic/glyoxysomal |
| Gh_A05G1787 | Receptor protein kinase | Gh_D04G0277 | oxoisovalerate dehydrogenase |
| Gh_A05G2095 | NAC domain-containing protein | Gh_D04G0970 | Hydrolases superfamily |
| Gh_A05G3515 | Myb domain protein | Gh_D05G1346 | Regulated G-protein |
| Gh_A06G0453 | hypothetical protein | Gh_D05G2238 | IAA-leucine |
| Gh_A06G0511 | Transmembrane | Gh_D05G2348 | [Gossypium arboreum] |
| Gh_A07G0243 | Cadherin | Gh_D05G3516 | PREDICTED: polyubiquitin-like |
| Gh_A07G2176 | F-box protein | Gh_D05G3813 | Vacuolar membrane-associated |
| Gh_A09G1158 | Guanylate kinase | Gh_D06G1453 | WRKY transcription factor |
| Gh_A09G1294 | zinc finger family | Gh_D08G0309 | facilitator superfamily |
| Gh_A09G1326 | AP2/B3-like transcriptional factor | Gh_D08G0600 | Uncharacterized protein |
| Gh_A10G1368 | Uncharacterized protein | Gh_D08G0850 | TBC1 domain family |
| Gh_A11G0161 | transmembrane receptor family | Gh_D08G1238 | Kinase family |
| Gh_A11G0260 | Chloroplast beta-amylase | Gh_D08G1987 | acetic acid-amido synthetase |
| Gh_A11G1892 | facilitator superfamily | Gh_D08G2033 | Cytochrome P450 |
| Gh_A12G1819 | UDP-Glycosyltransferase | Gh_D08G2151 | Ethylene-overproduction |
| Gh_A13G1419 | Vacuolar sorting-associated | Gh_D09G0981 | Chaperone |
| Gh_D01G0035 | Squalene synthase | Gh_D10G0363 | phosphatase-like protein |
| Gh_D01G0164 | F21B7.22 | Gh_D10G2073 | facilitator superfamily |
| Gh_D02G1362 | alpha-trehalose-phosphate synthase | Gh_D11G0279 | Chloroplast beta-amylase |
| Gh_D02G1573 | Rgs18 | Gh_D11G2958 | Kinase superfamily |
| Gh_D02G1987 | somatic embryogenesis kinase | Gh_D11G3342 | Hydroxymethylglutaryl-CoA lyase |
| Gh_D02G2145 | serine/threonine-protein kinase | Gh_Sca004885G02 | O-glucosyltransferase |

**Table S3.** GO terms related to ionic homeostasis

| **Term** | **ID** | **RSAS** | **LSAS** | **RSS** | **LSS** | **RAS** | **LAS** |  |
| --- | --- | --- | --- | --- | --- | --- | --- | --- |
| respiratory burst involved in  defense response | | GO:0002679 | 306 | 250 | 430 | 0 | 2 | 0 |
| response to chitin | GO:0010200 | 628 | 564 | 564 | 0 | 0 | 0 |  |
| response to cyclopentenone | GO:0010583 | 202 | 737 | 182 | 163 | 221 | 23 |  |
| response to water deprivation | GO:0009414 | 718 | 174 | 45 | 201 | 277 | 25 |  |
| cellular response to ethylene stimulus | GO:0071369 | 656 | 708 | 139 | 189 | 261 | 22 |  |
| response to chemical | GO:0042221 | 2480 | 2526 | 649 | 156 | 196 | 20 |  |
| cellular response to freezing | GO:0071497 | 666 | 708 | 425 | 237 | 27 | 30 |  |
| response to abscisic acid | GO:0009737 | 849 | 904 | 492 | 6 | 329 | 2 |  |
| response to salicylic acid | GO:0009751 | 39 | 591 | 444 | 4 | 80 | 0 |  |
| response to jasmonic acid | GO:0009753 | 7 | 1926 | 1637 | 237 | 5 | 29 |  |
| response to cold | GO:0009409 | 2536 | 4 | 1660 | 222 | 324 | 1 |  |
| response to oxygen-containing compound | GO:1901700 | 1924 | 2988 | 1686 | 71 | 30 | 8 |  |
| response to abiotic stimulus | GO:0009628 | 2131 | 2468 | 1409 | 72 | 78 | 21 |  |
| response to organic substance | GO:0010033 | 2660 | 0 | 4 | 84 | 266 | 0 |  |
| response to endogenous stimulus | GO:0009719 | 583 | 2112 | 1906 | 57 | 83 | 9 |  |
| response to stress | GO:0006950 | 3173 | 28 | 1271 | 59 | 61 | 7 |  |
| response to hormone | GO:0009725 | 3045 | 3128 | 0 | 34 | 30 | 1 |  |
| cellular response to fatty acid | GO:0071398 | 0 | 265 | 190 | 26 | 64 | 1 |  |
| phosphatidylinositol-mediated signaling | GO:0048015 | 260 | 2578 | 1973 | 13 | 80 | 3 |  |
| Total | | 22863 | 22659 | 15106 | 1831 | 2414 | 202 |  |

**Table S4. Primers used for qRT-PCR to validate the correlation with RNA-Seq**

| Gene ID | function annotation | Primers for qRT-PCR(5'-3') |
| --- | --- | --- |
| Gh_A12G2168 | Cys/Metmetabolism PLP-dependent enzyme | F:AGCTCTACCTACGTGCCTTG  R:CTGTTTAGGTGCCGAGGGTG |
| Gh_D11G0246 | LTP family | F:AGCGTGTTCAATCCAGGTAGC  R:ACAAAGAGCCAAGTTTTCCTGC |
| Gh_D06G1400 | Dormancy/auxin associated protein | F:AGCGTGTTCAATCCAGGTAGC  R:ACAAAGAGCCAAGTTTTCCTGC |
| Gh_D08G0875 | Sugar (and other) transporter | F:ATGATGTTCAGGCGGGTGG  R:TTGCTGTTTGCCAGTTCCGA |
| Gh_A01G0896 | Thaumatin family | F:CTGAACAAGAAGGAAGAGCGT  R:GCTTGTTCCTTGCACTCCTG |
| Gh_D10G1401 | Cytochrome P450 | F:GCTTGTGTCGGGATGACCTT  R:AACTGTGATACGAGGTGCGG |
| Gh_A12G0538 | Rhodanese-like domain | F:ACAAAGGCAGTTCTTGGAGGA  R:TGCCCTGCACTGAACTCTTC |
| Cotton_newGen4494 | Sulfite exporter TauE/SafE | F:GGCGGCATTTTTGTTCCAAT  R:TTCAAACGGCAGAGAGATAAGGA |
| Gh_A10G1465 | Late embryogenesis abundant protein | F:AGTTCCTTGGTGCTTCTGTT  R:TCGTTCCACTTTCCCCATCG |
| Gh_D04G1099 | Ubiquitin family | F:CCCAGATCAGCAAAGGCTCA  R:CACCACGGAGACGAAGAACA |
| Gh_D01G1177 | Raffinose synthase or seed imbibition protein Sip1 | F:GATCAGTTCTCCGCACCCAA  R:AACCAGCACCTTGGCAGTTA |
| Gh_D13G0029 | Ataxin-2 C-terminal region | F:ATGCCCCTCTGTTTGTTCCG  R:CTCGGTGTTGTCGTAAAAGCC |
| Gh_A08G0714 | Peroxidase | F:AGAAGGGATGGGTTCACAGC  R:GAGCACCAGACAAGGCTACT |
| Gh_D08G1956 | Glutathione peroxidase | F:CGATGTGAATGGTGAGAAGGC  R:GACATGGCCCTCCTTATCG |
| Gh_D06G1428 | Peptidase C1-like family | F:TGATTAATGTCCCGGATAGCATCG  R:GCCCAGCAACTTCCACAAGA |
| Gh_A02G1268 | Myo-inositol-1-phosphate synthase | F:ATTGTGTTTGGAGGATGGGACA  R:TTCCATGTAGGGTCTCAGTTGC |
| Gh_D02G0359 | PsaD | F:GGGGCAGAACTTTAGGAGCA  R:ACAACACTCATAAAGACCAGTAACA |
| Gh_D05G1742 | plastocyanin/azurin family | F:TCGCTATCCCATCTTTCACCG  R:TGACCCTTGGGACTGGAGAG |
| Gh_D07G2128 | RNA polymerase III subunit Rpc25 | F:GGGACTTCGGAGATGCAGAA  R:CATCGGGGCAAATGGTTTCG |
| Gh_D08G1408 | cyanobacterial aminoacyl-tRNA synthetase | F:GTCGATACCGGGGAGCTTTT  R:AGGCACTGAGTTAATGGCACT |

**
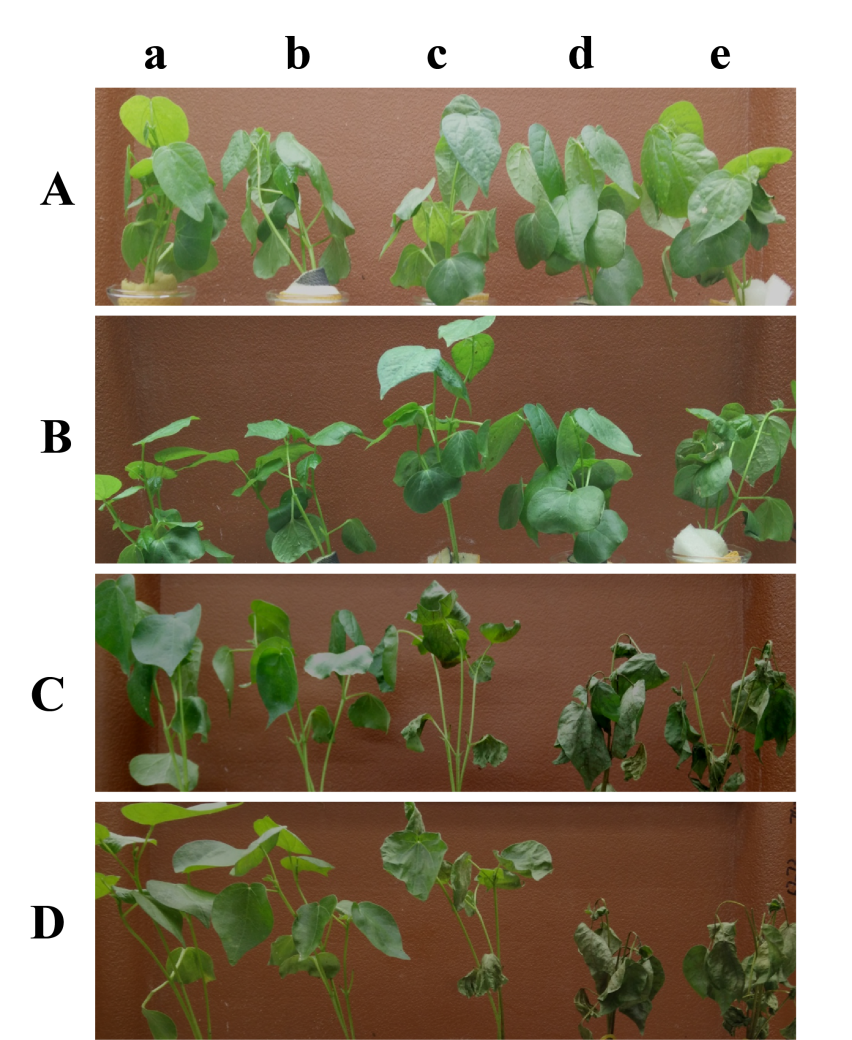
**

**Figure S1. Phenotypice changes in plants under different concentration of Na_2_CO_3_ and different times.** a-e: 0, 25, 50, 75, and 100 mM Na_2_CO_3_; A-D: after treat for 0 h, 6 h, 12 h, 24 h.


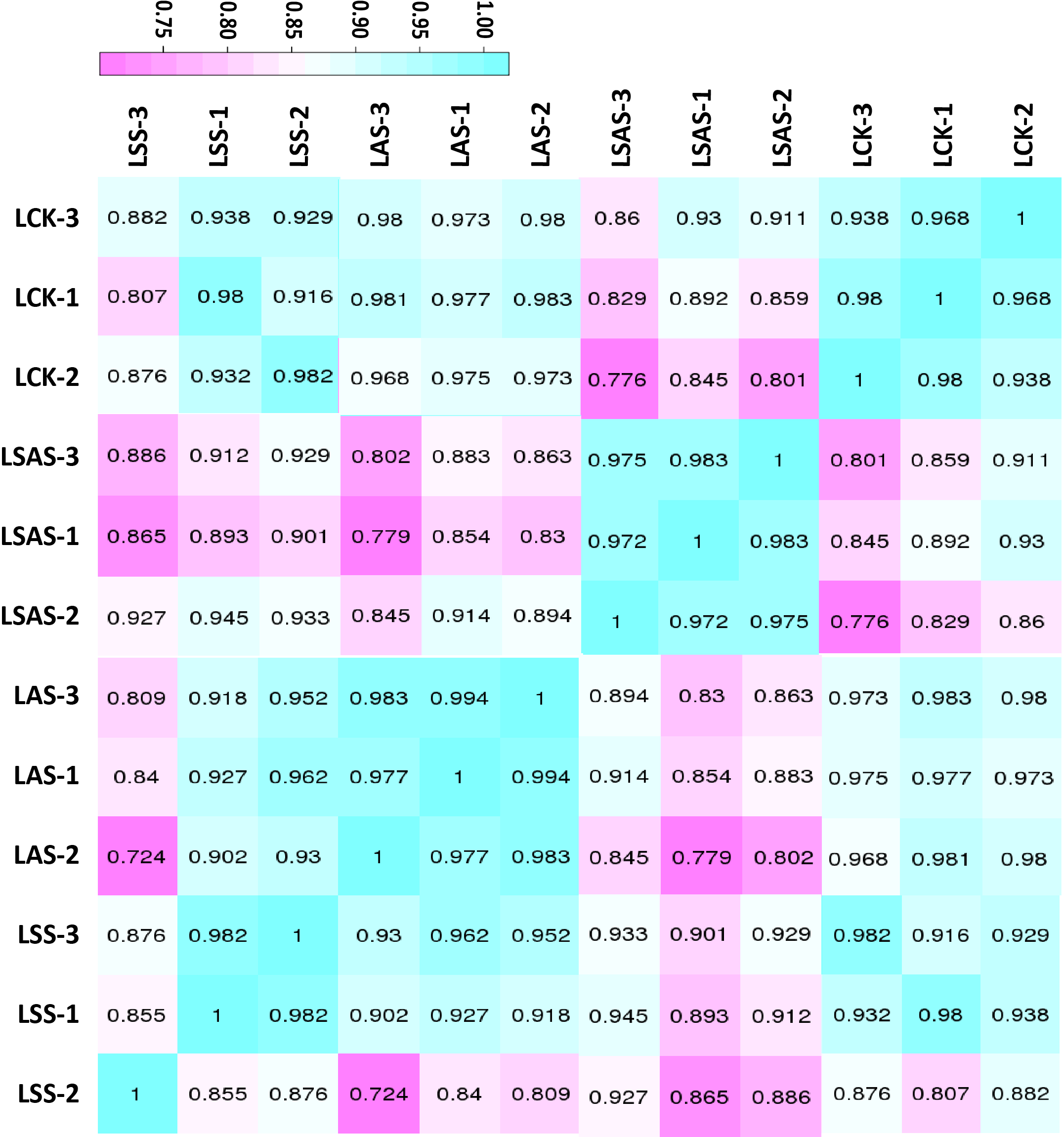


**Figure S2.** **Heatmap of Correlation of Expression Level among Leaf Samples.**Numbers in the box refer to Pearson’s correlation coefficient *r,* the colors of box represent the degree of correlation: blue represents high degree of correlation and pink represents low degree of correlation.


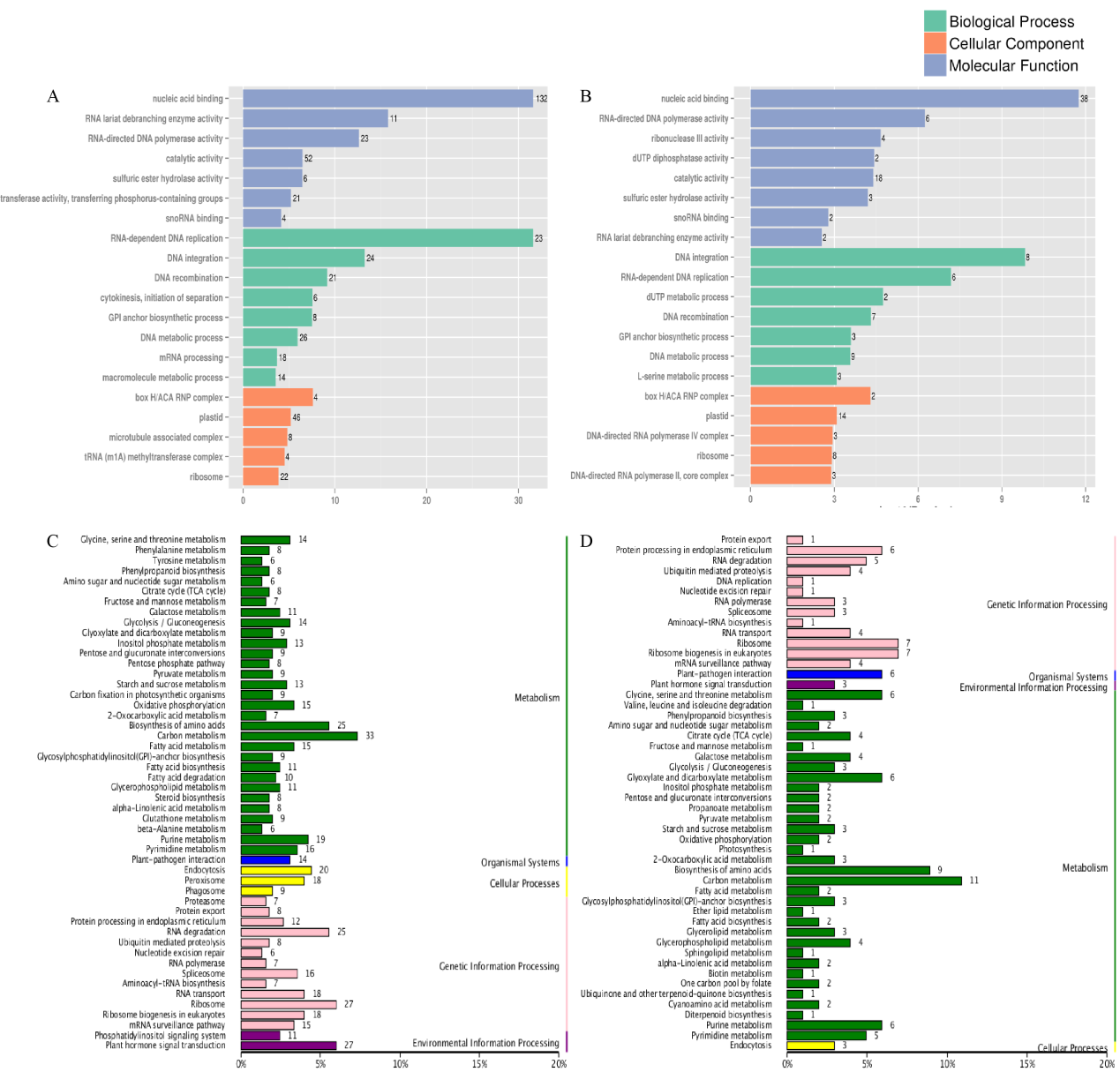


**Figure S3. GO and KEGG Enrichment of novel genes. (A)** GO analysis of novel genes in roots. **(B)** GO analysis of novel genes in leaves. **(C)** KEGG analysis of novel genes in roots. **(D)** KEGG analysis of novel genes in leaves.


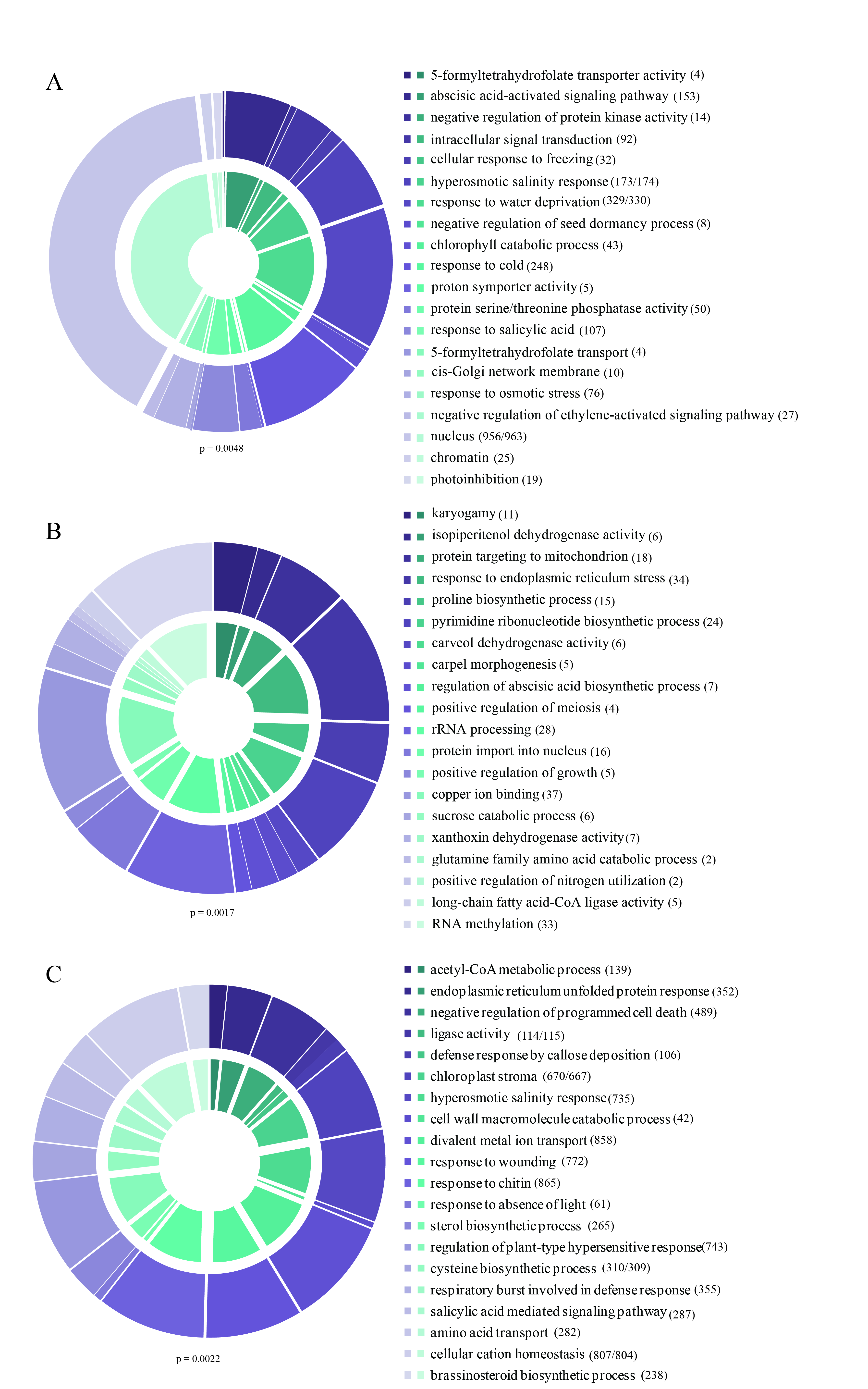


**Figure S4. GO enrichment analysis of DEGs.** The outer purple loop indicates the GO enrichment in leaves. The inner blue loop indicates the GO enrichment in roots. The size of the block indicates the DEG numbers. The numbers in the loops are threshold values of p-value. The colour of the block indicates the p-value of the GO term: a deeper colour represents a smaller p-value. (A) GO enrichment of DEGs under NaCl stress. (B) GO enrichment of DEGs under NaOH stress. (C) GO enrichment of DEGs under Na_2_CO_3_ stress.


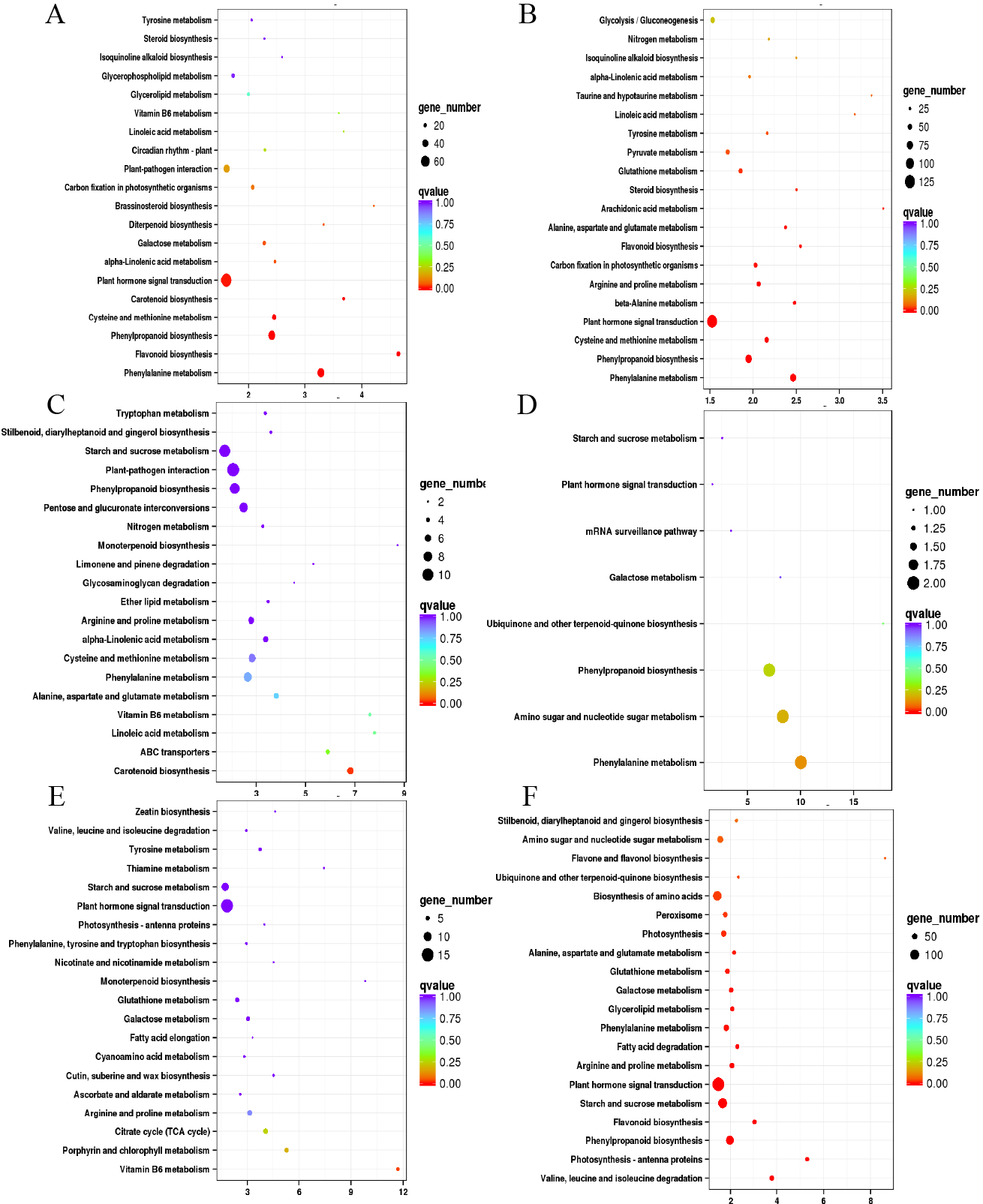


**Figure S5. KEGG Enrichment of DEGs.** Each circle represents the KEGG pathway X-axis represents the name og KEGG pathway; Y-axix represents enrichment factor that its size is positive correlation with enrichment-significance. a deeper color of the circle represents a smaller p- value which means more reliable of the enrichment. **(A)** KEGG analysis of DEGs in NaCl stress in roots. **(B)** KEGG analysis of DEGs in NaCl stress in leaves. **(C)** KEGG analysis of DEGs in NaOH stress in roots. **(D)** KEGG analysis of DEGs in NaOH stress in leaves. **(E)** KEGG analysis of DEGs in Na_2_CO_3_ stress in roots. **(F)** KEGG analysis of DEGs in Na_2_CO_3_ stress in leaves.


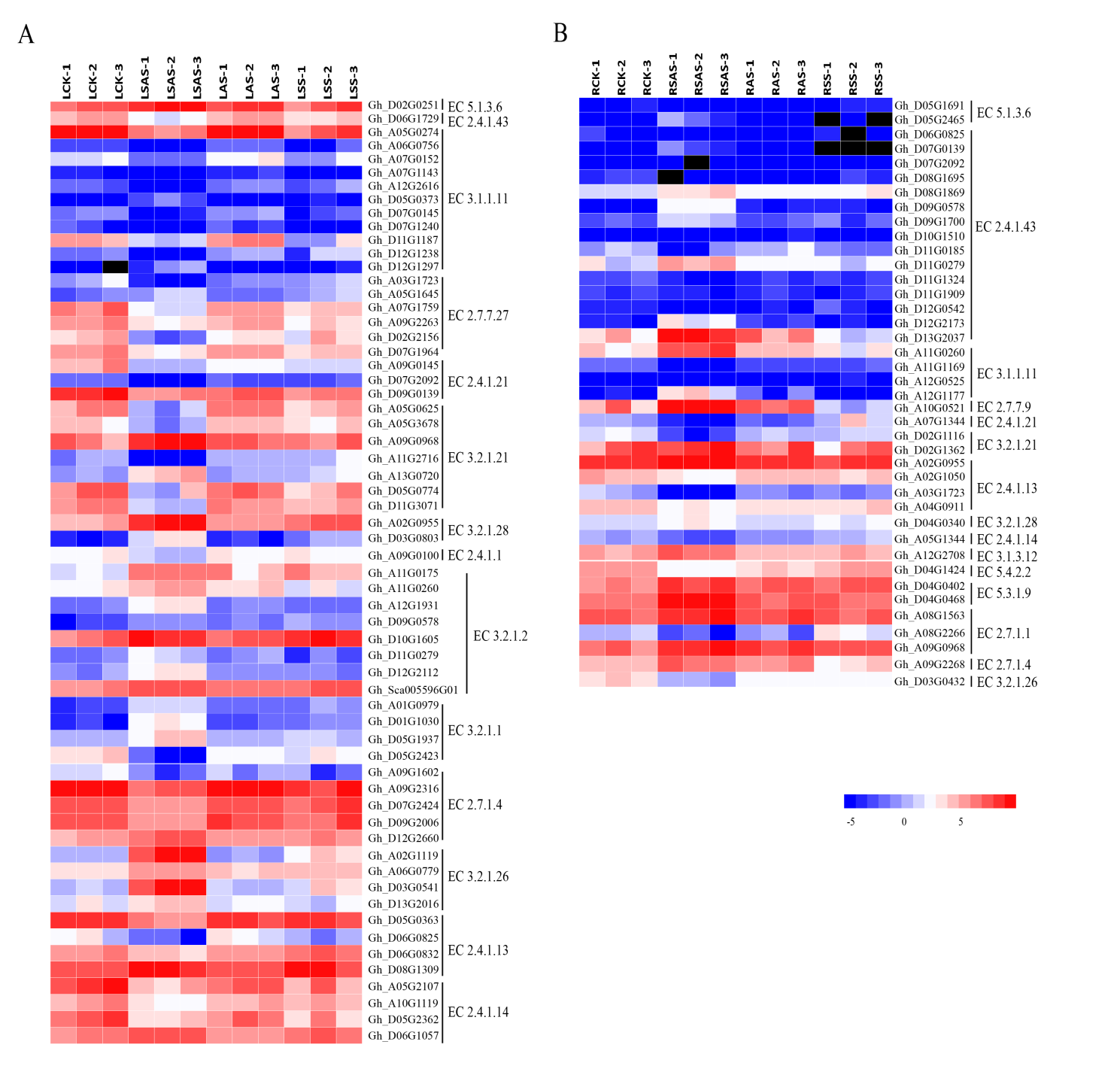


**Figure S6. Heatmap of sugar-related DEGs.** The right number of each heat map represents the number of enzyme. (A): Heatmap of leaf tissue DEGs (B): Heatmap of DEGs in root.

**
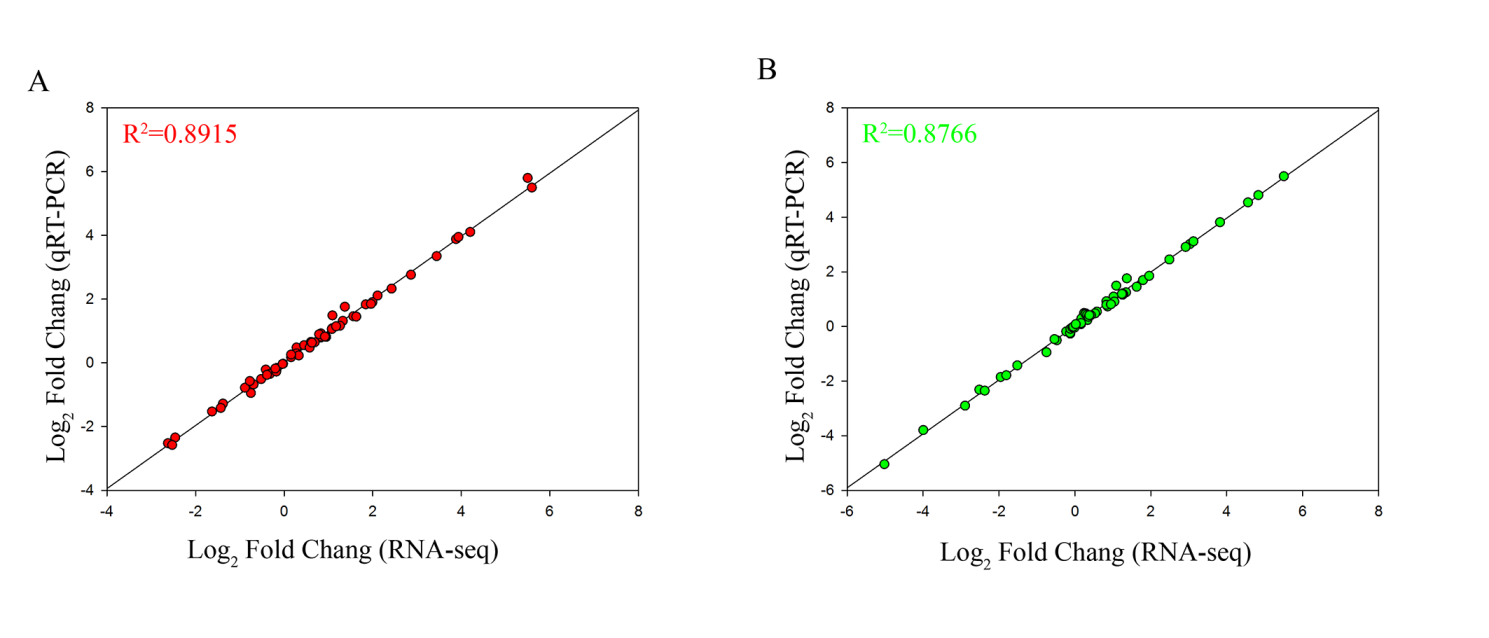
**

**Figure S7. qRT-PCR validation of the transcript levels of roots and leaves using RNA-seq under different treatments.** X-axis represents log_2_FC (Fold Change) derived from RNA-seq; Y-axis represents log_2_(2^-ΔΔCt^) specifically from the RT-qPCR experiment. (A) Transcript level of roots. (B) Transcript level of leaves.
